# Cell Membrane-Coated Nanotherapeutics for the Targeted Treatment of Acute and Chronic Colitis

**DOI:** 10.34133/bmr.0102

**Published:** 2024-11-07

**Authors:** Shan Li, Lei Chen, Tianyu Wu, Jingfeng Wu, Hong Yang, Qian Ju, Zhicheng Liu, Wensheng Chen, Dinglin Zhang, Yingxue Hao

**Affiliations:** ^1^ Army 953 Hospital, Shigatse Branch of Xinqiao Hospital, Army Medical University (Third Military Medical University), Shigatse, Tibet Autonomous Region 857000, China.; ^2^ Department of Chemistry, College of Basic Medicine, Army Medical University (Third Military Medical University), Chongqing 400038, China.; ^3^ Department of Gastroenterology, Southwest Hospital, Army Medical University (Third Military Medical University), Chongqing 400038, China.; ^4^ Department of Urology, Southwest Hospital, Army Medical University (Third Military Medical University), Chongqing 400038, China.; ^5^ Department of Vascular Surgery, Southwest Hospital, Army Medical University (Third Military Medical University), Chongqing 400038, China.

## Abstract

Integrin α4β1 and α4β7 are overexpressed in macrophages and leukocytes and play important roles in mediating cell homing and recruitment to inflammatory tissues. Herein, to enhance the targeting ability of nanotherapeutics for inflammatory bowel disease (IBD) treatment, cyclosporine A-loaded nanoparticles (CsA NPs) were coated with macrophage membranes (MM-CsA NPs) or leukocyte membranes (LM-CsA NPs). In vitro experiments demonstrated that the physicochemical properties of the nanotherapeutics (e.g., size, zeta potential, polymer dispersity index, and drug release profiles) did not obviously change after cell membrane coating. However, integrin α4β1 and α4β7 were expressed in MM-CsA NPs and LM-CsA NPs, respectively, which significantly inhibited normal macrophage phagocytosis and obviously increased uptake by proinflammatory macrophages and endothelial cells. In vivo experiments verified that cell membrane-coated nanotherapeutics have longer retention times in inflammatory intestinal tissues. Importantly, LM-CsA NPs significantly mitigated weight loss, alleviated colon shortening, decreased disease activity indices (DAIs), and promoted colon tissue repair in acute and chronic colitis model mice. Furthermore, LM-CsA NPs significantly decreased the expression of inflammatory factors such as TNF-α and IL-6 and increased the expression of gut barrier-related proteins such as E-cadherin, ZO-1, and occludin protein in colitis mice.

## Introduction

Inflammatory bowel disease (IBD) is an immune-mediated chronic gastrointestinal disease, including Crohn’s disease (CD) and ulcerative colitis (UC) [1,2]. IBD is usually observed in adolescents and young adults, which is easy to recur and difficult to treat [3]. The typical symptoms of IBD include vomiting, bloody diarrhea, abdominal pain, weight loss, fatigue, and fever, which seriously affect the quality of life of patients [4]. If IBD patients do not receive timely treatment, the risk of intestinal cancer rapidly increases over time, ultimately leading to the development of small intestine and colon cancer [5]. Although the pathogenesis of IBD is still not fully understood, it is widely believed that genetic susceptibility, immune disorders, gut microbiota dysbiosis, mucosal barrier dysfunction, and environmental factors are responsible for the occurrence of IBD [6].

The current goal of IBD treatment is to control mucosal inflammation to prevent disease progression and complications [7]. Aminosalicylic acid drugs, glucocorticoids, immunomodulators, and other biologics have all been successfully used in the clinical treatment of IBD [8–10]. However, the adverse effects of small-molecule drugs and the instability of biotherapeutics in vivo present challenges for IBD treatment. To address these issues, nanodrug delivery systems have been used to deliver therapeutics for the targeted treatment of IBD [10–12]. In addition, low pH values and high concentrations of reactive oxygen species (ROS) have been found in the microenvironment of IBD tissues [13–16]. Based on the abnormal microenvironment, pH- and ROS-responsive nanodrug delivery systems have been developed for the targeted delivery of therapeutics for IBD treatment [17,18]. Although controlled release of therapeutics at inflammatory sites in the intestines has been achieved, the ability of nanotherapeutics to target inflamed intestinal tissue needs to be further investigated. Cell membrane-coated nanotherapeutics retain the biological functions of source cells and are widely used to increase the targeting ability and therapeutic efficacy of nanotherapeutics [19,20].

During IBD flare-ups, leukocytes are recruited from the circulation to the intestinal mucosa to perform immune functions [21]. In this process, both integrins expressed by leukocytes and cell adhesion molecules (CAMs) mediate the adhesion and migration of leukocytes [22]. Integrin α4β1 and α4β7 play important roles in the regulation of leukocyte homing and recruitment to inflammatory tissues [23]. Integrin α4β1 is expressed in most leukocytes, and its main ligand is vascular cell adhesion molecule-1 (VCAM-1), which is mainly expressed in endothelial cells after cytokine stimulation [22]. Integrin α4β7 is increased on immunoglobulin A (IgA)-secreting plasma cells, memory T cells, and activated intestinal homing CD4^+^ T cells [24], and its main ligand is mucosal address cell adhesion molecule-1 (MadCAM-1). In addition, VCAM-1 is also a ligand of integrin α4β7 [25]. It has been demonstrated that the expression of MadCAM-1 and VCAM-1 is up-regulated in the colonic mucosae of patients with IBD as well as in animal models [26,27]. Consequently, integrin α4β7-overexpressing leukocyte cell membranes have been used to coat nanotherapeutics for the targeted treatment of IBD [28]. In addition, integrin α4β1 was found to be overexpressed on the macrophage membrane (MM); consequently, MM-coated nanotherapeutics have also been used for the targeted treatment of IBD [29,30].

**Fig. 1.**
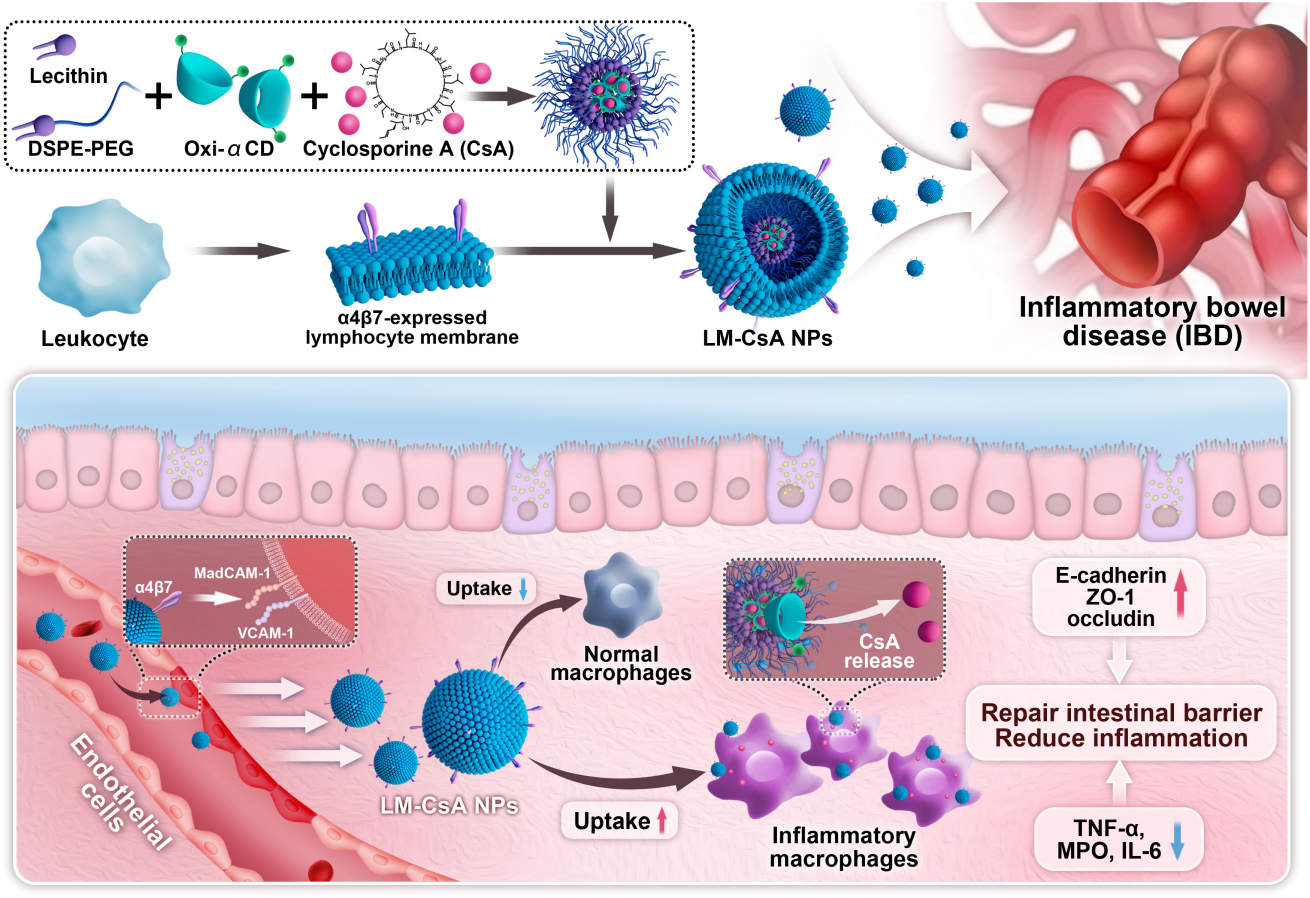
Fabrication of cell membrane-coated CsA NPs for IBD treatment.

Our team previously developed ROS- or pH/ROS-responsive nanodrug delivery systems to effectively deliver programmed death ligand-1 or cyclosporine A (CsA), respectively, to the intestinal tissue for IBD treatment [17,18]. Herein, to enhance the targeting ability of nanotherapeutics, CsA-loaded ROS-responsive nanoparticles (CsA NPs) were coated with integrin α4β1-overexpressing MMs (MM-CsA NPs) or α4β7-overexpressing lymphocyte membranes (LM-CsA NPs) for the targeted treatment of IBD (Fig. 1). In vitro experiments demonstrated that α4β1 and α4β7 were expressed on MM-CsA NPs and LM-CsA NPs, respectively. Both cell membrane-coated NPs, especially LM-CsA NPs, were successfully internalized by activated macrophages and avoided phagocytosis by normal macrophages. In vivo experiments demonstrated that both MM-CsA NPs and LM-CsA NPs exhibited longer retention times in inflammatory intestinal tissues than that of CsA NPs. Furthermore, cell membrane-coated nanotherapeutics displayed greater efficacy in treated colitis mice than noncoated CsA NPs.

## Materials and Methods

### Materials and reagents

4-(Hydroxymethyl) phenylboronic acid pinacol ester-modified cyclodextrin (ROS-responsive α-CD material, oxi-αCD) was synthesized, characterized, and prepared by our laboratory [31]. Cy5 carboxylic acid and 1,2-distearoyl-sn-glycero-3-phosphoethanolamine-*N*-methoxy (polyethylene glycol)-2000 (DSPE-PEG_2000_) were obtained from Xi’an Ruixi Corporation (Xi’an, China). Lecithin was purchased from Alfa Aesar (Ward Hill, MA, USA). Pluronic F127 and 4′,6-diamidino-2-phenylindole (DAPI) were purchased from Sigma-Aldrich LLC (St Louis, MO, USA). CsA was obtained from J&K Scientific Co. (Beijing, China). Dextran sulfate sodium (DSS) was purchased from Meilun Bio (Dalian, China).

Fetal bovine serum (FBS), Dulbecco’s modified Eagle’s medium (DMEM), and RPMI 1640 medium were obtained from Boster Biological Technology Co. (Wuhan, China). CCK-8 reagent and enhanced chemiluminescence (ECL) solution were obtained from Baoguang Biotechnology Co. (Chongqing, China). Anti-interleukin-6 (IL-6) and anti-tumor necrosis factor-α (TNF-α) antibodies were purchased from Affinity Biosciences (Changzhou, China). Anti-E-cadherin, anti-occludin and anti- ZO-1 antibodies were purchased from Sanying Biotechnology Co. (Wuhan, China). Anti-β1 and anti-β7 antibodies were purchased from Bioss Bio. (Beijing, China). Anti-α4β7 and secondary antibodies were purchased from Cell Signaling Technology Inc. (Boston, MA, USA). A membrane and cytosol protein extraction kit, phenylmethanesulfonyl fluoride (PMSF), Coomassie blue staining solution, lipopolysaccharide (LPS), and DiO (3,3′-dioctadecyloxacarbocyanine perchlorate, a green fluorescent probe of cell membrane) were obtained from Beyotime Biotechnology (Shanghai, China). A myeloperoxidase (MPO) assay kit was purchased from Nanjing Jingcheng Bioengineering Institute (Nanjing, China).

The mouse colon cancer cell line Mc 38.2 was obtained from the American Type Culture Collection (Rockville, MD, USA). Mouse monocyte macrophages (RAW 264.7 cells) and human umbilical vein endothelial cells (HUVECs) were obtained from the Cell Bank of the Chinese Academy of Sciences (Shanghai, China). Mouse T lymphoma cells (TK-1 cells) were purchased from Mingzhou Biotechnology Co. (Ningbo China). Male C57 mice (6 weeks old, 22 ± 2 g) were purchased from Gempharmatech Co. (Nanjing, China).

### Preparation of cell membrane vesicles

MMs were isolated from RAW 264.7 cells, and lymphocyte membranes (LMs) were obtained from TK-1 cells (a type of lymphocyte with α4β7 overexpression) according to previously reported methods [32,33]. Cell membrane vesicles were obtained with a cell membrane protein extraction kit. Briefly, the collected cells were dispersed in a membrane protein extraction buffer solution supplemented with PMSF for 15 min and homogenized on ice approximately 30 times. The cell suspension was centrifuged at 800*g* for 10 min (4 °C) and 14,000*g* for 30 min (4 °C). The precipitate was resuspended in ultrapure water. The extracted cell membrane suspension was sonicated for 15 min (42 kHz, 100 W) followed by repeated extrusion 15 times through a 400-nm polycarbonate porous membrane using a mini-liposome extruder (Avestin, Canada) to obtain cell membrane vesicles. The collected vesicles were stored in water at 4 °C.

### Fabrication of CsA NPs

CsA-loaded NPs were fabricated using a nanoprecipitation/self-assembly method [31]. In brief, 6.0 mg of lecithin and 6.0 mg of DSPE-PEG_2000_ were dispersed in 400 μl of anhydrous ethanol and 10.0 ml of deionized water. The mixture was gently stirred (120 rpm) at 65 °C for 30 min. In parallel, 5.0 mg of CsA and 50.0 mg of oxi-αCD were dissolved in 2.0 ml of methanol. The solution was slowly added dropwise into the preheated lipid dispersion (1.0 ml/min). The mixture was stirred rapidly for 3 min (800 rpm) and then slowly stirred for 2 h at room temperature (80 rpm). The NPs were collected by centrifugation at 15,000 rpm for 10 min, washed with 5% F127 (0.4 ml) and ultrapure water (10 ml) 3 times, and then resuspended in 0.2 ml of ultrapure water. Cy5-labeled NPs were prepared by a similar method except that 5.0 mg of Cy5-labeled oxi-αCD was used.

### Fabrication and characterization of MM-CsA NPs and LM-CsA NPs

MMs and LMs were coated on CsA NPs by direct extrusion. Briefly, cell membrane vesicles and CsA NPs were mixed with a membrane protein-to-polymer mass ratio of 1:1 and dispersed by ultrasound (42 kHz, 100 W) for 3 min. Then, the mixture was extruded through a 200-nm polycarbonate porous membrane 10 times using a small liposome extruder to obtain MM-CsA NPs and LM-CsA NPs.

The particle size, polydispersity index (PDI), and zeta potential of the NPs were measured by dynamic light scattering (DLS) and laser Doppler anemometry using a Malvern Zetasizer (Nano ZS, Malvern, UK). To observe the morphology of the NPs, freshly prepared MM-CsA NPs and LM-CsA NPs were diluted 200-fold with ultrapure water and added dropwise to a copper grid. The dried NPs were stained with 5% phosphotungstic acid, and their morphology was observed by a transmission electron microscope (JEM-1400, Japan). The stability of the NPs was determined by measuring the particle size and PDI of the NPs in ultrapure water at different time points.

The concentration of CsA was quantified by high-performance liquid chromatography (HPLC). HPLC was performed using a C18 column (250 mm × 4.6 mm, 5 μm) with a 210-nm detection wavelength. The mobile phase consisted of acetonitrile and water (87:13, v/v) with a flow rate of 1.2 ml/min and a column temperature of 70 °C. To investigate the in vitro drug release profiles of the NPs, 150 μl of newly prepared MM-CsA NP or LM-CsA NP suspension was added to a dialysis tube (molecular weight cutoff: 3,500 Da) and immersed in 40 ml of phosphate-buffered saline (PBS) (pH 7.34) or PBS containing 1.0 mM H_2_O_2_ at 37 °C. At the indicated time points, 4 ml of the supernatant was removed for HPLC analysis, and another 4.0 ml of fresh solution was added. The cumulative release percentage was calculated according to the concentration of CsA measured by HPLC.

### Protein analysis of MM-CsA NPs and LM-CsA NPs

The proteins of MM, LM, MM-CsA NPs, LM-CsA NPs, RAW 264.7 cells, and TK-1 cells were extracted and analyzed by sodium dodecyl sulfate–polyacrylamide gel electrophoresis (SDS-PAGE). Coomassie blue solution was used for gel staining, followed by decolorization overnight. The gel was imaged using a protein imaging system. The expression of integrin α4 and β1 in RAW 264.7 cells, MM cells, and MM-CsA NPs and the expression of integrin α4 and β7 in TK-1 cells, LM cells, and LM-CsA NPs were detected by Western blotting.

### Fluorescence colocalization

Cy5-labeled CsA NPs were coated with DiO-labeled MMs or LMs to prepare double-labeled MM-CsA NPs and LM-CsA NPs, respectively. RAW 264.7 cells were seeded in 12-well plates at a density of 2 × 10^5^ cells/well overnight. Double fluorescence-labeled MM-CsA NPs and LM-CsA NPs with a final concentration of 1 μg/ml Cy5 were added to the plate. After incubation for 6 h, the plates were fixed with 4% paraformaldehyde for 20 min at room temperature, and the nuclei were stained with DAPI. Confocal laser scanning microscopy (CLSM) (Carl Zeiss LSM 780, Germany) was used to observe the fluorescent signals.

### Cell uptake

RAW 264.7 cells and HUVECs were seeded into 12-well cell culture plates at a density of 2 × 10^5^ cells per well overnight. Then, the cells were treated with or without 1 μg/ml LPS for 12 h. Subsequently, free Cy5 or Cy5-labeled NPs were added to the medium and incubated for 4 h. Finally, the cells were fixed with 4% paraformaldehyde, and the nuclei were stained with DAPI. Fluorescent signals were observed by using CLSM. Integrin inhibition experiments were performed by adding DATK32 (MedChemExpress, Shanghai, China) and BIO-1211 (InvivoChem, Guangzhou, China) at a concentration of 1 μg/ml to RAW 264.7 cells and HUVECs, respectively, following incubation with Cy5-labeled NPs4 h for CLSM imaging.

### Cytotoxicity evaluation

RAW 264.7 cells and Mc 38.2 cells were seeded in 96-well plates at a density of 1.0 × 10^4^ cells per well overnight, and different concentrations of CsA (0.6, 1.25, 2.5, 5, 10, and 20 μg/ml) and NPs were added into each well and incubated with cells for 4 and 24 h. Subsequently, 10 μl of CCK-8 solution was added to each well and cultured for another 2 h at 37 °C. The absorbance at 450 nm was determined using a microplate reader (Thermo Fisher Scientific), and cell activity was calculated based on absorbance.

### In vitro anti-inflammation experiments

RAW 264.7 cells were uniformly seeded into 6-well cell culture plates at a density of 1 × 10^6^ cells per well overnight. Before treatment, the cells were treated with 1 μg/ml LPS for 6 h. Then, 10 μg/ml of free CsA, CsA NPs, MM-CsA NPs, or LM-CsA NPs was added to the medium and incubated for 24 h. The expression of IL-6 was detected after protein extraction by Western blotting.

### Generation of a colitis mouse model

Male C57 mice (6 weeks, 22 ± 2 g) were purchased from Gempharmatech Co. Ltd. (Nanjing, China). All animal experiments were performed following guidelines approved by the Army Medical University Ethics Committee (Chongqing, China). To establish the acute colitis model, the mice were fed standard chow and treated with 3% (w/v) DSS for 7 consecutive days. The DSS-supplemented drinking water was changed every 2 to 3 days. The chronic colitis model was constructed by administering drinking water supplemented with 1.5% (w/v) DSS for 7 days followed by a period of drinking normal drinking water for 14 days. This 21-day cycle was repeated 3 times. Age-matched healthy receiving tap water served as controls. Disease activity indices (DAIs), including body weight, rectal bleeding, and stool consistency (Table S1), were assessed daily after colitis induction [34].

### In vivo biodistribution

To assess the biodistribution of NPs and colon targeting, mice with acute colitis were randomly divided into 4 groups (*n* = 18 per group, 3 mice at each of time points). Saline, free Cy5, Cy5-labeled CsA NPs, Cy5-labeled MM-CsA NPs, or LM-CsA NPs (1 mg/kg Cy5) were injected into model mice via the tail vein. The mice were sacrificed at 2, 6, 12, 24, 48, and 72 h after injection, and their colons were dissected. In vitro imaging was performed with an in vivo imaging system (IVIS) (PerkinElmer; Waltham, MA, USA). After 24, 48, and 72 h, the colons from the CsA NP, MM-CsA NP, and LM-CsA NP groups were fixed in 4% paraformaldehyde and cut into 10-μm frozen sections. CLSM was used to observe the distribution and intensity of fluorescence in colon tissue after nuclear staining with DAPI.

### In vivo therapeutic efficacy

Healthy mice without any treatment served as controls. Mice with DSS-induced colitis were randomly divided into 5 groups (*n* = 6). For the acute colitis model, on day 4 after DSS induction, the mice were administered saline, free CsA, CsA NPs, MM-CsA NPs, or LM-CsA NPs (equivalent to 2 mg/kg/day of CsA) via the tail vein for 3 consecutive days. The mice were sacrificed on day 10. For the chronic colitis model, mice were given saline, free CsA, CsA NPs, MM-CsA NPs, or LM-CsA NPs (equivalent to 2 mg/kg/day of CsA) via the tail vein on the fourth day of each DSS induction cycle. On day 10 of the last cycle of DSS induction, the mice were sacrificed.

When the mice were sacrificed, blood was collected to evaluate the levels of hematological indicators, including alanine transaminase (ALT), aspartate transaminase (AST), and creatinine (Cr). The remaining colon tissue was fixed in 4% paraformaldehyde for hematoxylin and eosin (H&E), immunohistochemical, and immunofluorescence staining. Major organs (e.g., heart, liver, spleen, lung, kidney, and testis) were collected, and the spleen was weighed to calculate the spleen index. All organs were then fixed in 4% paraformaldehyde for histological examination.

### Endoscopic analysis of colitis

Colonoscopy was used to observe the severity of colitis in the mice at the end of the experiment. The murine endoscopic index of colon severity (MEICS) was used to quantify the severity of colitis in mice [35]. Thickening of the colon wall, changes in the normal vascular pattern, presence of fibrin, mucosal granularity, and stool consistency of the mice were scored from 0 to 3, and the MEICS was the final cumulative score of the abovementioned 5 parameters (Table S2).

### Histological staining

The colon and other major organs (*n* = 5) of the mice were fixed in 4% paraformaldehyde for 24 h, dehydrated, cleared, embedded in paraffin, and then cut into 3-μm-thick sections. These sections were stained with H&E. Tissue sections were scored according to the extent of inflammatory cell infiltration into the colon, crypt damage, ulceration, and edema [36]. The scoring criteria are shown in Table S3.

### Western blot analysis

Cells and tissues were lysed in radioimmunoprecipitation assay buffer. The total amount of protein was determined by the bicinchoninic acid (BCA) method. The protein samples were electrophoresed on SDS-polyacrylamide gels and transferred to polyvinylidene fluoride (PVDF) membranes. Protein bands were detected with antibodies against α_4_ (1:2,000), β_1_ (1:2,000), β_7_ (1:2,000), TNF-α (1:2,000), IL-6 (1:2,000), glyceraldehyde-3-phosphate dehydrogenase (GAPDH) (1:3,000), and β-tubulin (1:3,000). Goat anti-rabbit horseradish peroxidase was used as a secondary antibody. The protein bands were visualized using an ECL solution and a chemiluminescence imaging system.

### MPO level detection

The colonic tissue was homogenized in normal saline, and MPO levels were detected according to the instructions of the MPO assay kit.

### Immunofluorescence staining

The expression levels of E-cadherin and ZO-1 were detected by immunofluorescence staining. Sections were deparaffinized using xylene and a graded alcohol series, followed by antigen retrieval using sodium citrate. Then, the sections were blocked with 5% goat serum and incubated overnight with anti-E-cadherin (1:2,000) and anti-ZO-1 (1:2,000) antibodies at 4 °C. The sections were washed 3 times with PBS and then incubated with secondary antibodies. Finally, the sections were stained with DAPI before sealing. Images of the sections were captured using a fluorescence microscope.

### Immunohistochemical staining

The expression levels of occludin were detected by immunohistochemical staining. The sections were first deparaffinized using xylene and a graded alcohol series, followed by antigen retrieval using sodium citrate. The sections were then treated with 3% hydrogen peroxide and blocked with goat serum. The samples were then incubated with anti-occludin (1:100) antibodies and horseradish peroxidase-conjugated secondary antibodies, followed by DAB (diaminobenzidine) and hematoxylin staining. After dehydration and sealing, the samples were imaged with a microscope.

### Statistical analysis

Statistical analyses were performed using GraphPad Prism version 9.0 software (GraphPad, USA). One-way and 2-way analyses of variance (ANOVAs) and *t* tests were used to determine statistical significance (*P < 0.05, **P < 0.01, ***P < 0.001, and ****P< 0.0001). All the data are presented as the means ± SDs. All the detailed statistical analysis results are listed in the Supplementary Materials (Tables S4 to S28).

## Results

### Fabrication and characterization of NPs

**Fig. 2.**
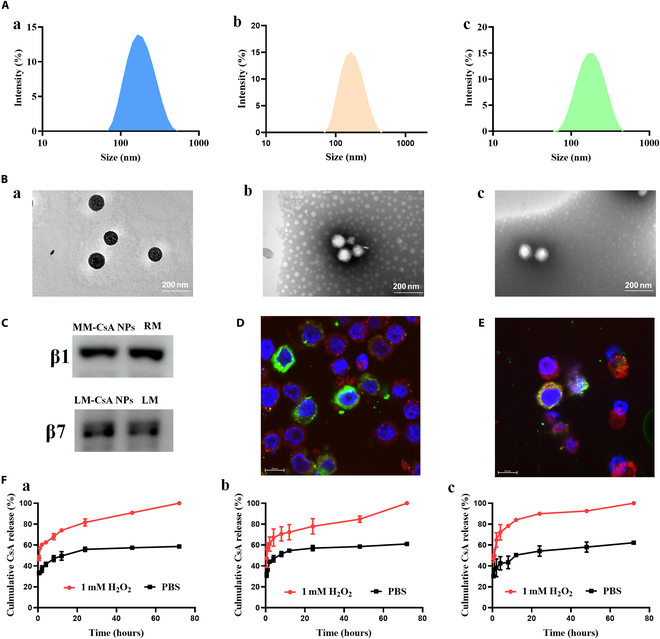
(A and B) Size distributions (A) and TEM images (B) of CsA NPs (a), MM-CsA NPs (b), and LM-CsA NPs (c). (C) Expression of β1 and β7 in cell membrane-encapsulated NPs and the cell membrane. (D and E) CLSM images of the colocalization of Cy5-labeled NPs (red) with DiO-labeled MMs (D) and lymphocyte membranes (LM) (E) (green) in RAW 264.7 cells. The cell nucleus was stained with DAPI (blue). (F) Drug release of CsA from CsA NPs (a), MM-CsA NPs (b), and LM-CsA NPs (c) incubated with or without 1.0 mM H_2_O_2_ in PBS.

First, CsA-loaded ROS-responsive NPs (CsA NPs) were prepared. The particle size of the CsA NPs was 155 ± 0.4 nm, the PDI was 0.21 ± 0.01, the zeta potential was −24.4 ± 1.1 mV, and the drug loading and encapsulation efficiencies were 9.59 ± 0.04% and 25.90 ± 0.10%, respectively (Table). DLS revealed that the CsA NPs had a uniform size distribution (Fig. 2Aa), and transmission electron microscopy (TEM) revealed that the CsA NPs were spherical in shape (Fig. 2Ba). These results suggest that the CsA NPs were successfully fabricated.

**Table.** Physicochemical characterization of various nanoformulations

Nanoformulations	Size (nm)	PDI	Zeta potential (mV)
CsA NPs	155.4 ± 0.4	0.21 ± 0.01	−24.4 ± 1.1
MM	242.8 ± 4.2	0.32 ± 0.04	−38.9 ± 0.8
MM-CsA NPs	174.3 ± 1.9	0.26 ± 0.03	−29.3 ± 1.5
LM	187.4 ± 14.8	0.30 ± 0.03	−30.8 ± 0.6
LM-CsA NPs	169.4 ± 2.9	0.22 ± 0.01	−27.0 ± 3.8

MMs and LMs were successfully obtained from RAW 264.7 cells and TK-1 cells, and the surfaces of CsA NPs were coated with the cell membrane vesicles by direct extrusion. The size, PDI, and zeta potential of the membrane vesicles and membrane-coated CsA NPs are listed in Table. Compared to those of CsA NPs, the particle sizes of MM-CsA NPs and LM-CsA NPs slightly increased to 174 ± 2 nm and 169 ± 3 nm, respectively (Table). Interestingly, compared to those of MMs (243 ± 4) and LMs (187 ± 15 nm), the sizes of MM-CsA NPs and LM-CsA NPs were decreased (Table). In addition, the zeta potentials of the MM-CsA NPs and LM-CsA NPs were slightly lower than that of the CsA NPs (Table), which may be helpful for increasing the stability of the NPs [37]. DLS results indicated that both the MM-CsA NPs and LM-CsA NPs had suitable size distributions (Fig. 2Ab and c). TEM images revealed “core-shell” structures in both the MM-CsA NPs and LM-CsA NPs. The reason is that the cell membrane has a bimolecular lipid layer structure, which forms the NP shell.

SDS-PAGE was used to verify the protein profiles of the cell membrane-coated NPs. As shown in Fig. S1, the typical protein bands of macrophages and TK-1 cells were found in MMs, MM-CsA NPs, LMs, and LM-CsA NPs. Integrin α4β1 and α4β7 on the macrophage and leukocyte surfaces are responsible for leukocyte homing and recruitment to inflammatory tissues by binding to CAMs during the development of IBD [23]. Consequently, MM- or LM-coated NPs can directly target inflammatory tissues via α4β1 or α4β7. Western blot analysis was used to verify the expression of integrins in MM-CsA NPs and LM-CsA NPs. As shown in Fig. 2C, MMs and MM-CsA NPs expressed integrin β1, while LMs and LM-CsA NPs expressed integrin β7. These results confirmed that the MM- and LM-coated NPs retained their functional proteins, which is beneficial for enhancing the targeting of the NPs to inflammatory sites.

To further verify whether the NPs were successfully coated with the cell membranes, the CsA NPs were coated with DiO-labeled MMs and LMs. These dual-fluorescence probe-labeled NPs were incubated with RAW 264.7 cells and visualized by CLSM. As shown in Fig. 2D and E, the red fluorescence of the CsA NPs and the green fluorescence of the cell membrane appeared at the same location near the nucleus of the RAW 264.7 cells. These results further demonstrated that CsA NPs were successfully coated with MMs and LMs.

To study the stability of the NPs, freshly prepared NPs were incubated in ultrapure water. The size and PDI of the NPs were determined at various time points. As shown in Fig. S2A and B, the particle size and PDI of CsA NPs, MM-CsA NPs, and LM-CsA NPs did not change significantly in ultrapure water after 48-h incubation.

The CsA release profiles from NPs treated with or without H_2_O_2_ were determined. As expected, CsA was released more rapidly from the CsA NPs in the 1.0 mM H_2_O_2_ group than in the PBS group (Fig. 2Fa). Interestingly, the drug release profiles of the MM-CsA NPs and LM-CsA NPs were similar to those of the CsA NPs (Fig. 2Fb and c), suggesting that the cell membrane coating did not alter the drug release profile of the NPs.

In summary, the TEM and CLSM results confirmed that the MM and LM-coated CsA NPs were successfully prepared. Western blotting results demonstrated that MM-CsA NPs and LM-CsA NPs retained the functional proteins of the MMs and LMs, respectively. In addition, the cell membrane coating did not obviously change the physicochemical properties of the CsA NPs.

### In vitro cytotoxicity and anti-inflammatory effect of NPs

RAW 264.7 cells and Mc 38.2 cells were used to assess the potential toxicity of the NPs. As shown in Fig. S2C, the viability of the Mc 38.2 cells slightly decreased with increasing CsA concentration. In addition, the viability of the Mc 38.2 cells in the MM-CsA NP and LM-CsA NP groups did not significantly differ from that in the CsA and CsA NP groups (Fig. S2C). In contrast, the viability of the RAW 264.7 cells decreased with increasing CsA concentration (Fig. S2D). Notably, the viability of RAW 264.7 cells decreased dramatically with high concentrations of CsA after 24 h of incubation (Fig. S2D). However, the viability of the RAW 264.7 cells treated with CsA NPs, MM-CsA NPs, or LM-CsA NPs remained over 50% after 24 h of incubation (Fig. S2D), indicating that NP formation decreased the toxicity of CsA. In addition, there was no significant difference among the CsA NP, MM-CsA NP, and LM-CsA NP groups, suggesting that the cell membrane coating did not change the biosafety of the CsA NPs.

IL-6 plays an important role in promoting tissue injury, activation of acute phase reaction, autoimmune reaction, and metabolism [38]. Consequently, the pro-inflammatory factor IL-6 was used to assess the anti-inflammatory ability of NPs. As shown in Fig. S3A and B, compared to the control group, IL-6 expression was markedly increased in RAW 264.7 and HUVECs with LPS treatment. However, IL-6 expression was decreased obviously with CsA NP, MM-CsA NP, or LM-CsA NP treatment (Fig. S3A and B). Importantly, LM-CsA NPs decreased IL-6 expression obviously compared to other groups (Fig. S3A and B). These results indicated that the cell membrane-coated NPs have stronger anti-inflammatory activity than CsA or CsA NPs.

### Cellular uptake of NPs

NPs are easily ingested by phagocytes such as monocytes and macrophages in the reticuloendothelial system (RES) [39], which removes NPs from the body. Biomimetic NPs can prevent phagocytosis by phagocytes [19]. Herein, RAW 264.7 and HUVECs were used to assess the cellular uptake ability of NPs.

As shown in Fig. S4A, compared to free Cy5, Cy5-labeled CsA NPs, Cy5-labeled MM-CsA NPs, and LM-CsA NPs exhibited obvious red fluorescence after 2 and 4 h of incubation, suggesting that Cy5-labeled CsA NPs can be easily internalized by RAW 264.7 cells. Importantly, the fluorescence signals in the Cy5/MM-CsA NP and Cy5/LM-CsA NP groups were significantly lower than those in the Cy5/CsA NP group after 2 and 4 h of incubation (Fig. S4A), indicating that the cell membrane coating can decrease the phagocytosis of NPs by macrophages. The semiquantitative analysis of fluorescence intensity demonstrated that there was a significant difference between the Cy5/CsA NP group and the Cy5/MM-CsA NP or Cy5/LM-CsA NP group after 2 and 4 h of incubation (Fig. S4B). After 4 h of incubation, the fluorescence intensity in the Cy5/LM-CsA NP group was significantly lower than that in the Cy5/MM-CsA NP group (Fig. S4B), indicating that LM coating can effectively prevent the phagocytosis of CsA NPs by macrophages.

LPS is usually used to induce inflammatory macrophages. As expected, the fluorescence intensities in the Cy5/CsA NP, Cy5/MM-CsA NP, and Cy5/LM-CsA NP groups were greater than those in the non-LPS treatment group after 2 or 4 h of incubation (Fig. S4C), suggesting that the phagocytosis of NPs by proinflammatory macrophages was increased. Interestingly, compared with those in the Cy5/CsA NP group, the Cy5/MM-CsA NP and Cy5/LM-CsA NP groups displayed stronger fluorescence signals after 4 h of incubation (Fig. S4C). Importantly, the Cy5/LM-CsA NP group exhibited stronger fluorescence than the Cy5/MM-CsA NP group, indicating that cell membrane coating can enhance the internalization of NPs by proinflammatory macrophages. This increased internationalization may be as a result of the remaining proteins on the surface of the biomimetic NPs increasing the affinity for proinflammatory macrophages. In addition, flow cytometry and semiquantitative analysis also demonstrated that the phagocytosis of MM- and LM-coated NPs by RAW 264.7 cells was decreased, while that of the MM- and LM-coated NPs by proinflammatory macrophages was increased (Fig. S5A to C). Similarly, the LM coating effectively prevented the internalization of the NPs by RAW 264.7 and enhanced the NPs to proinflammatory macrophages (Fig. S5A to C).

When NPs are administered by intravenous injection, they inevitably come into contact with endothelial cells (e.g., HUVECs). Consequently, the cellular uptake of Cy5-labeled NPs by control or LPS-treated HUVECs was observed by CLSM. As shown in Fig. S6A and B, both the Cy5/MM-CsA NP and Cy5/LM-CsA NP groups displayed stronger fluorescence intensity in control or LPS-treated HUVECs than the Cy5/CsA NP group, indicating that cell membrane-coated NPs can be easily internalized by HUVECs. In addition, with LPS stimulation, the ability of HUVECs to phagocytose NPs was enhanced. The reason may be that activated endothelial cell surfaces up-regulate several adhesion molecules including E-selectin, intercellular adhesion molecule (ICAM)-1, p-selectin, and VCAM-1 [40], which is helpful for leukocyte recruitment. After LPS treatment, HUVECs were activated and the expression of receptors on the surface increased, which led to increased uptake of the membrane-encapsulated NPs. These results confirmed that inflammatory endothelial cells have significantly increased uptake of membrane-encapsulated NPs, which helps them migrate from the blood to sites of inflammation in colonic tissue. In addition, LPS-stimulated RAW 264.7 cells and HUVECs treated with inhibitory antibodies against integrins significantly reduced cellular uptake of NPs (Fig. S7A to C), which further confirmed that integrin α4β1 and α4β7 play important roles in the cellular uptake of cell membrane-coated NPs.

### Biodistribution of NPs

**Fig. 3.**
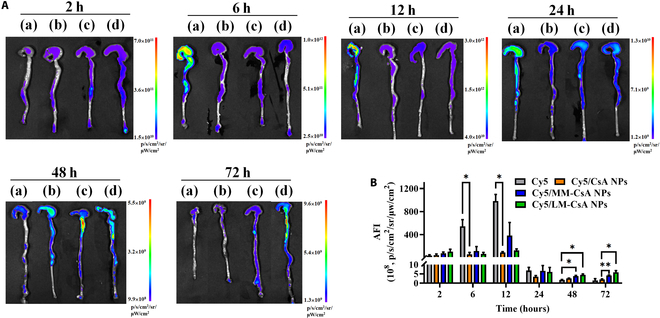
Biodistribution of NPs in the colons of DSS-induced colitis mice. (A) In vitro fluorescence images of mouse colons at different time points after administration of free Cy5 (a), Cy5/CsA NPs (b), Cy5/MM-CsA NPs (c), and Cy5/LM-CsA NPs (d). (B) Region of interest analysis of fluorescence intensity in the colons at different time points. *, significantly different at *P* < 0.05; **, significantly different at *P* < 0.01.

Free Cy5 and Cy5-labeled NPs were injected into mice with DSS-induced acute colitis via the tail vein, and the colon and major organs were imaged in vitro and subjected to semiquantitative analysis of fluorescence intensity. As shown in Fig. 3A, the fluorescence signals of the colon in the free Cy5 group reached a maximum at 12 h after administration and were obviously attenuated at 24 h after injection. Similar results were obtained for the Cy5/CsA NP, Cy5/MM-CsA NP, and Cy5/LM-CsA NP groups (Fig. 3A). However, after 48 h and 72 h of treatment, considerable fluorescence signals were observed in the colon tissues of mice in the Cy5/MM-CsA NP and Cy5/LM-CsA NP groups (Fig. 3A), suggesting that the MM and LM coatings can increase the retention time of NPs in colon tissues. Semiquantitative analysis also revealed stronger fluorescence intensity in the Cy5/MM-CsA NP and Cy5/LM-CsA NP groups than in the uncoated NP group at 24, 48, and 72 h after administration (Fig. 3B). CLSM was used to observe the aggregation of NPs in the colon tissue of mice after 24, 48, and 72 h of administration. The results also demonstrated that MM and LM coatings can prolong the retention time of NPs in colon tissues after 72 h of treatment (Fig. S8A and B). In addition, the fluorescence intensity of major organs in mice showed that Cy5-labeled MM-CsA NPs and LM-CsA NPs accumulated predominantly in the liver, spleen (Fig. S9A and B), and lung, because more phagocytes were found in these organs. After 72 h of administration, fluorescence signals were still observed in small intestine, indicating that cell membrane coating prolonged retention time in small intestine. Collectively, our results demonstrated that MM and LM coatings can enhance the targeting ability and prolong the retention time of NPs in inflamed colon tissue.

### In vivo efficacies of NPs on acute colitis model mice

**Fig. 4.**
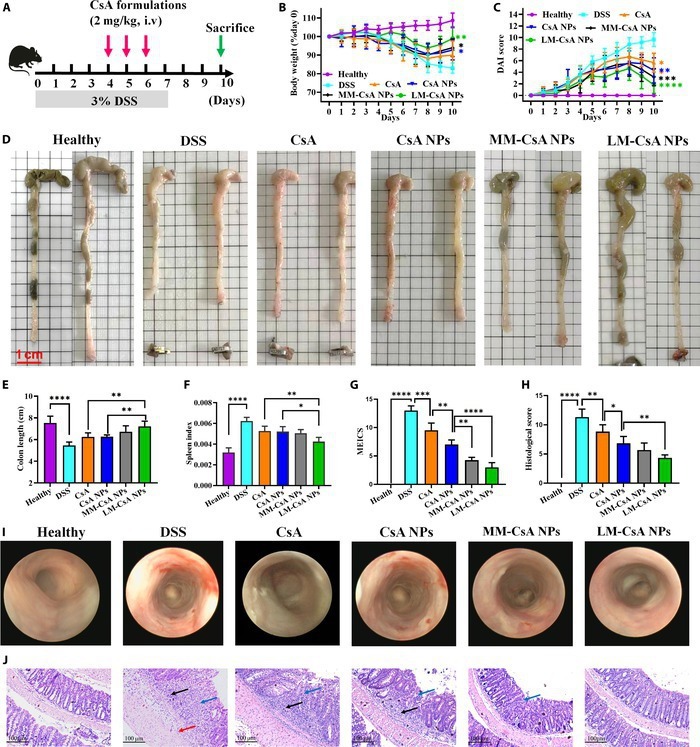
In vivo therapeutic efficacy of NPs in DSS-induced acute colitis mice. (A) DSS and NP administration regimens in the acute mouse colitis model. (B and C) Body weights (B) and DAIs (C) of acute colitis model mice. (D and E) Representative macroscopic image of a colon (D) and analysis of colon length (E) on day 10. (F) Spleen index on day 10. (G) Analysis of MEICSs of mice in different groups. (H) Analysis of the histological score of colonic tissues. (I) Representative endoscopic image. (J) Representative H&E-stained histological sections. Red arrows indicate mucosal edema, black arrows indicate inflammatory cell infiltration, and blue arrows indicate crypt damage. *, significantly different at *P* < 0.05; **, significantly different at *P* < 0.01; ***significantly different at *P* < 0.001; ****significantly different at *P* < 0.0001.

To evaluate the efficacy of the NPs, an acute colitis mouse model was established (Fig. 4A). During the experiment, body weight, fecal characteristics, and rectal bleeding were recorded every day, and the DAI was calculated. As shown in Fig. 4B and C, mice in the DSS group exhibited significantly reduced body weights and markedly elevated DAIs, demonstrating that the acute colitis mouse model was successfully established. Compared to those in the DSS group, body weight loss and DAIs were lower in the CsA, CsA NP, MM-CsA NP, and LM-CsA NP groups (Fig. 4B and C). In addition, compared with acute colitis model mice treated with free CsA, model mice treated with CsA NPs or cell membrane-coated CsA NPs exhibited less body weight loss and decreased DAIs. Importantly, LM-CsA NP-treated mice had the lowest DAI and highest body weight compared to those of mice in other groups (Fig. 4B and C), suggesting that CsA NPs encapsulated by cell membranes, especially LMs, can effectively improve the symptoms of colitis model mice.

The length of the colon is considered an important indicator of colonic inflammation. As shown in Fig. 4D and E, the colon length significantly decreased after 3% DSS treatment. The colon length of the mice in each treatment group was increased, and the increase in colon length in the MM-CsA NP and LM-CsA NP groups was the most obvious (Fig. 4D and E). Importantly, the colon length of the LM-CsA NP group was significantly longer than that of the other groups (Fig. 4D and E). Compared with those in normal mice, the spleen volume and weight in colitis mice increased significantly (Fig. 4F). The spleen indices of the mice treated with LM-CsA NPs were significantly lower than those of the CsA- and CsA-NP-treated mice.

Colonoscopy was used to observe the colonic mucosa of the mice on the last day of the experiment, and the MEICS was determined according to the endoscopic results. As shown in Fig. 4I, the colonic mucosa of healthy mice was smooth, whereas the mucosa of colitis mice was rough, with loss of vascular texture, erosive bleeding, and ulceration. The mucosa of the CsA and CsA NP groups was still rough, with extensive erosion and redness (Fig. 4I). In the MM-CsA NP group, lesions in the colonic mucosa were obviously alleviated (Fig. 4I). Interestingly, the colonic mucosa of the LM-CsA NP group was smooth and was not significantly different from that of the normal group (Fig. 4I). In addition, the MEICS significantly increased after DSS induction (Fig. 4G). However, the MEICS decreased dramatically in mice treated with CsA and its nanoformulations (Fig. 4G). Importantly, the MEICS of mice in the MM-CsA NP and LM-CsA NP groups was significantly lower than that of mice in the CsA NP group. These results suggested that cell membrane-coated NPs significantly relieve intestinal mucosal inflammation and injury.

The colonic tissues were stained with H&E and scored histologically. As shown in Fig. 4J, the colonic tissues of DSS-induced mice showed marked mucosal edema, loss of goblet cells, and destruction of crypt swelling, with extensive inflammatory cell infiltration in the mucosa and submucosa, as well as epithelial cell damage. After treatment, the extent of colonic injury in the CsA group decreased to some extent (Fig. 4J). In the CsA NP and MM-CsA NP groups, a few inflammatory cells infiltrated the crypts (Fig. 4J). The colonic epithelium of the LM-CsA NP group was intact, which was similar to that of the normal group (Fig. 4J). To further evaluate recovery from colonic injury, histological scoring was performed. As shown in Fig. 4H, the histological scores of the colon in the CsA group were lower than those in the DSS group but were significantly greater than those in the CsA NP group. Both MM-CsA NPs and LM-CsA NPs reduced the histological score of the colon, but LM-CsA NPs achieved better therapeutic effects than CsA NPs (Fig. 4H). These results further demonstrated that compared with uncoated CsA NPs, LM-CsA NPs can significantly promote colonic tissue repair and reduce intestinal inflammation.

**Fig. 5.**
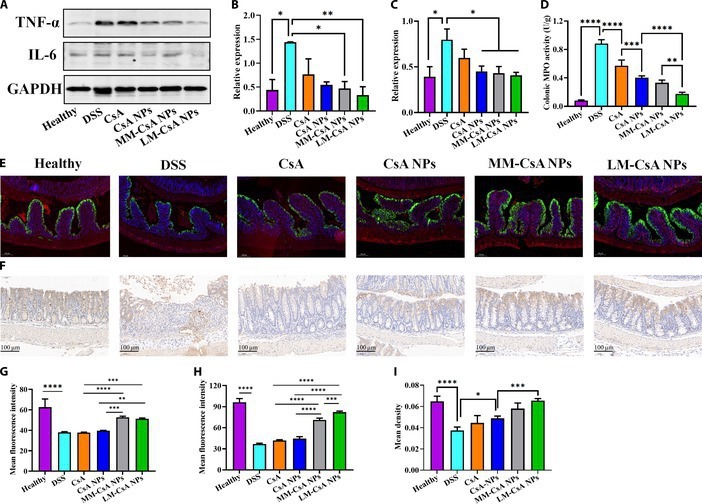
Detection of the levels of inflammatory factors, MPO, and colon barrier-associated proteins in the colons of mice with acute colitis. (A) TNF-α, IL-6, and GAPDH levels in colon tissue were measured by Western blotting. (B and C) Relative expression of TNF-α (B) and IL-6 (C). (D) MPO levels in colon tissue. (E) Immunofluorescence results for ZO-1 and E-cadherin in the colonic tissue of mice with acute colitis (*n* = 4). Red, ZO-1; green, E-cadherin; blue, DAPI (nucleus). (F) The protein expression of occludin in the colon tissues of mice with acute colitis was detected by immunohistochemistry (*n* = 4). (G to I) Statistical analysis of ZO-1 (G), E-cadherin (H), and occludin protein (I) expression levels. *, significantly different at *P* < 0.05; **, significantly different at *P* < 0.01; ***significantly different at *P* < 0.001; ****significantly different at *P* < 0.0001.

To explore the anti-inflammatory mechanism of cell membrane-coated MM-CsA NPs and LM-CsA NPs, the expression levels of inflammatory factors in the mouse colon were analyzed by Western blotting. As shown in Fig. 5A to C, the levels of TNF-α and IL-6 were increased in the colonic tissues of mice after DSS induction. Compared with free CsA and CsA NPs, the MM-CsA NPs and LM-CsA NPs significantly decreased the expression of TNF-α and IL-6 (Fig. 5A to C). We also examined the levels of MPO in the colonic tissues of the mice. After DSS induction, the levels of MPO in the colons of the mice were significantly increased (Fig. 5D). As expected, compared with those in the colonic tissues of mice in the CsA NP and MM-CsA NP groups, the MPO levels in the colonic tissues of mice in the LM-CsA NP group were obviously lower (Fig. 5D). These results suggested that LM-CsA NPs may play an anti-inflammatory role by decreasing inflammatory factor and MPO levels.

According to previous findings, dysfunction of the intestinal epithelial mechanical barrier is a characteristic pathological change in UC [41]. Tight junction proteins and E-cadherin play important roles in the maintenance of the intestinal epithelial barrier. Increased permeability of tight junctions directly promotes the progression of UC [42]. Tight junctions are composed of multiple protein complexes, mainly occludin, claudin-1, and ZO-1 [43]. We examined the expression of E-cadherin, ZO-1, and occludin in tight junctions using immunohistochemistry and immunofluorescence. As shown in Fig. 5E, E-cadherin and ZO-1 were fluorescently costained in the colonic tissues, and E-cadherin was clearly expressed on one side of the villi of the luminal colon (green fluorescence). ZO-1 was widely distributed in the colonic mucosa between cells (red fluorescence). However, the expression of E-cadherin and ZO-1 in the colonic tissues of DSS-induced mice decreased significantly (Fig. 5E and Fig. S10). Semiquantitative analysis indicated that the expression levels of E-cadherin and ZO-1 in the MM-CsA NP and LM-CsA NP groups were significantly greater than those in the CsA and CsA NP groups (Fig. 5G and H). The expression of E-cadherin in the LM-CsA NP group was significantly greater than that in the MM-CsA NP group (Fig. 5H). We performed immunohistochemical staining for occludin protein in colonic tissues (Fig. 5F). Semiquantitative analysis verified that occludin protein expression was significantly decreased in the colonic tissues of mice after DSS induction and was significantly increased after treatment with CsA or its nanoformulations (Fig. 5I). LM-CsA NPs were superior to other therapeutics at restoring occludin protein expression (Fig. 5I). These results suggested that LM-CsA NPs can repair the intestinal barrier and promote the healing of the colonic mucosa in mice with acute colitis.

### In vivo efficacies of NPs on chronic colitis

**Fig. 6.**
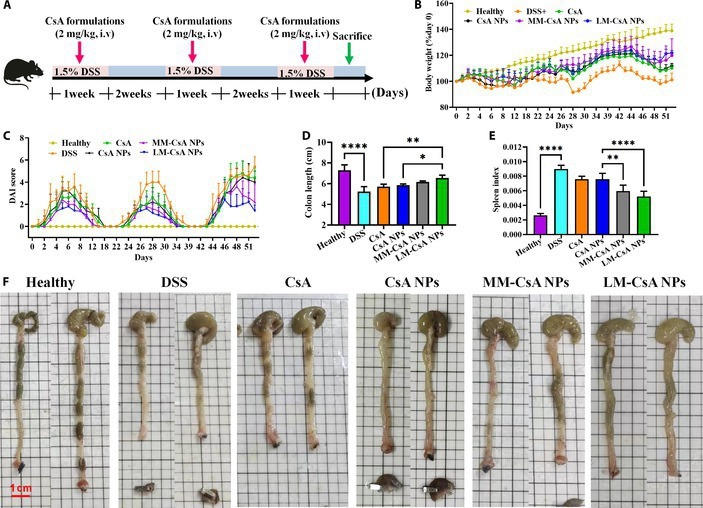
In vivo therapeutic efficacy of NPs in DSS-induced chronic colitis model mice. (A) DSS and NP administration regimens in the chronic mouse colitis model. (B and C) Body weights (B) and DAIs (C) of acute colitis model mice. (D and E) Analysis of colon length (D) and the spleen index (E) at the end of the experiment. (F) Representative macroscopic image of the colon. *, significantly different at *P* < 0.05; **, significantly different at *P* < 0.01; ****, statistically significantly different at *P* < 0.0001.

The establishment and treatment of chronic colitis mice are described in Fig. 6A. As shown in Fig. 6B and C, the body weights and DAIs of the mice in the treatment group fluctuated periodically over the course of the dosing cycle. Compared with those in the DSS group, the body weight percentages of the mice in each treatment group were significantly increased (Fig. 6B). At the end of treatment, the MM-CsA NP and LM-CsA NP groups had significantly greater body weight percentages than the CsA and CsA NP groups (Fig. 6B). The DAIs in the MM-CsA NP and LM-CsA NP groups were lower than those in the other groups (Fig. 6C). The DAIs of the mice in the LM-CsA NP group were significantly lower than those in the CsA and CsA NP groups (Fig. 6C). However, the MM-CsA NP group did not significantly differ from the CsA and CsA NP groups in terms of body weight or DAI (Fig. 6C). These results suggested that the LM coating can effectively increase the therapeutic efficacy of CsA NPs in the treatment of chronic colitis.

Compared with that in normal mice, colonic length in chronic colitis model mice was markedly shorter, which was associated with long-term exposure to chronic inflammatory stimuli. As shown in Fig. 6F, colon length increased in all treatment groups at the end of the experiments. Interestingly, compared with CsA and CsA NPs, LM-CsA NPs significantly restored colon length (Fig. 6D). The spleen indices of the mice in the MM-CsA NP and LM-CsA NP groups were obviously lower than those in the CsA NP group, but the decrease was more pronounced in the LM-CsA NP group (Fig. 6E). These results further demonstrated that the cell membrane coating increased the therapeutic efficacy of CsA NPs in chronic colitis model mice.

**Fig. 7.**
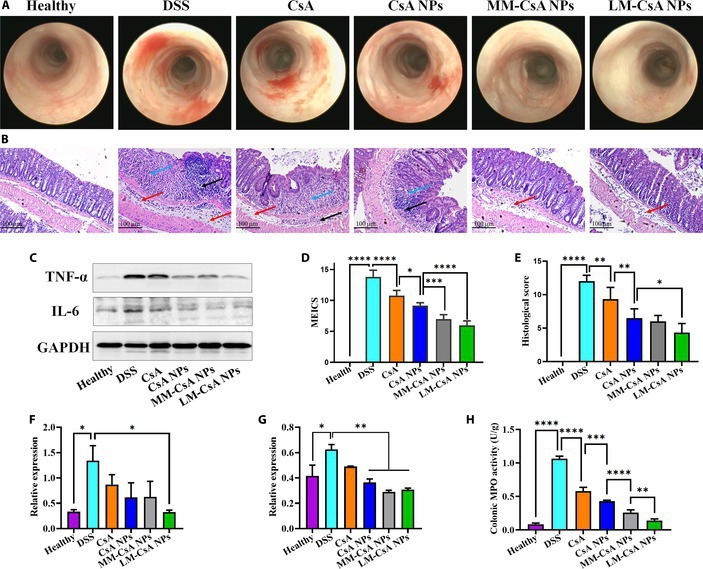
Endoscopic analysis, histological evaluation, and proinflammatory cytokine levels in chronic colitis model mouse tissue. (A) Representative endoscopic image on the last day of the experiment. (B) Representative H&E-stained histological sections. Red arrows indicate mucosal edema, black arrows indicate inflammatory cell infiltration, and blue arrows indicate crypt damage. (C) TNF-α, IL-6, and GAPDH levels in colon tissue were measured by Western blotting. (D) Analysis of MEICSs. (E) Analysis of the histological score of colonic tissues. (F and G) Relative expression of TNF-α (F) and IL-6 (G). (H) MPO levels in colon tissue. *, significantly different at *P* < 0.05; **, significantly different at *P* < 0.01; ***, significantly different at *P* < 0.001; ****, significantly different at *P* < 0.0001.

In addition, the colonic mucosa of chronic colitis model mice had severe damage, hyperemia, and edema, with extensive ulceration and diffuse distribution of lesions (Fig. 7A). The injury to the colonic mucosa of the mice in the CsA group was not obvious (Fig. 7A). In the CsA NP group, the colonic mucosa was rough with congestion and edema (Fig. 7A). The colonic mucosa was slightly rough with little fibrin attachment in the MM-CsA NP group (Fig. 7A). Importantly, the colonic mucosa was smooth, with only local congestion and redness in the LM-CsA NP group (Fig. 7A). In addition, the MM-CsA NP and LM-CsA NP groups had significantly lower MEICSs than the CsA NP group (Fig. 7D). These results suggested that membrane-coated CsA NPs can alleviate mucosal damage in chronic colitis model mice and promote the repair of the colonic mucosa.

The colon tissues of the mice in each group were also cut into sections and stained with H&E. In the DSS group, the colonic mucosa was markedly thickened, goblet cells and crypts were absent, and inflammatory cell infiltration was extensive (Fig. 7B). The CsA group still exhibited severe inflammation in the colon, loss of crypts and goblet cells, and the presence of small ulcers (Fig. 7B). In the CsA NP group, the inflammation of colon tissue was reduced, and crypt destruction was mitigated (Fig. 7B). Both the MM-CsA NP and LM-CsA NP groups had an intact colonic epithelium, obvious goblet cells and crypts, and limited infiltration of inflammatory cells (Fig. 7B). Compared with those in the CsA group, the histological colonic scores in the CsA NP group were significantly lower (Fig. 7E). However, the histological scores of the LM-CsA NP group were significantly lower than those of the CsA NP group (Fig. 7E). In conclusion, compared with uncoated CsA NPs, LM-CsA NPs significantly ameliorated the colonic histological damage caused by colitis in mice and promoted the resolution of inflammation and repair of the colonic mucosa.

IL-6 and TNF-α, important mediators of chronic intestinal inflammation, were also examined by Western blotting (Fig. 7C). Similar to that in the colonic tissues of acute colitis model mice, IL-6 and TNF-α expression was significantly increased in the colonic tissues of chronic colitis model mice (Fig. 7F and G). Similarly, MM-CsA NPs and LM-CsA NPs significantly decreased the expression of IL-6 in colonic tissues, while the expression of TNF-α decreased only in the LM-CsA NP group compared to the DSS group (Fig. 7F and G). In addition, the LM-CsA NPs significantly reduced MPO level in the colonic tissues compared to that in the other groups (Fig. 7H). These results suggested that LM-CsA NPs may play an anti-inflammatory role by reducing the levels of inflammatory factors and MPO in chronic colitis model mice.

**Fig. 8.**
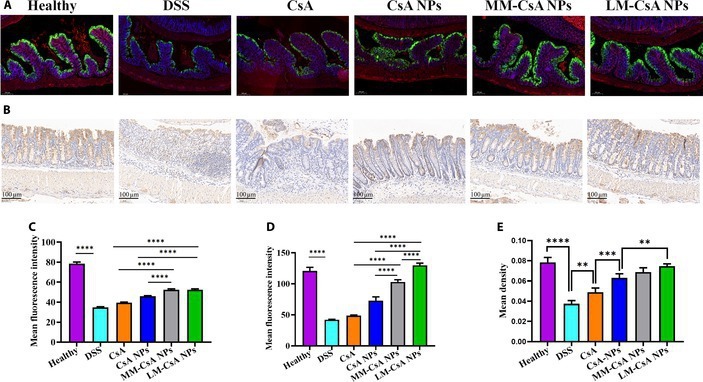
Detection of the levels of colon barrier-associated proteins in the colon tissues of mice with chronic colitis. (A) Immunofluorescence results for ZO-1 and E-cadherin in the colonic tissue of mice with chronic colitis (*n* = 4). Red, ZO-1; green, E-cadherin; blue, DAPI (nucleus). (B) The expression of occludin protein in the colon tissue of mice with chronic colitis was detected by immunohistochemistry (*n* = 4). (C to E) Statistical analysis of ZO-1 (C), E-cadherin (D), and occludin protein (E) expression levels. **, significantly different at *P* < 0.01; ***, significantly different at *P* < 0.001; ****, significantly different at *P* < 0.0001.

H&E staining showed that chronic colitis appeared to be more severe as a result of the functional disruption of the intestinal barrier (Fig. 7B). The protein expression of E-cadherin and ZO-1 in colonic tissues was detected by immunofluorescence (Fig. 8A and Fig. S11). Compared to those in the colonic tissues of normal mice, the levels of the E-cadherin (green fluorescence) and ZO-1 proteins (red fluorescence) were significantly decreased in the colonic tissues of mice after DSS induction. The expression of E-cadherin and ZO-1 in colonic tissues was increased after treatment with CsA and its nanoformulations. Semiquantitative analysis demonstrated that compared to CsA and CsA NPs, MM-CsA NPs and LM-CsA NPs significantly increased E-cadherin and ZO-1 expression in the colonic tissues of mice (Fig. 8C and D). Compared to that in acute colitis model group, the expression of ZO-1 in the MM-CsA NP and LM-CsA NP groups was not significantly different, but the expression of E-cadherin in the LM-CsA NP group was significantly greater than that in the MM-CsA NP group (Fig. 8C and D). We also used immunohistochemistry to stain occludin proteins. As shown in Fig. 8B, occludin protein expression was significantly decreased in the colonic tissue of chronic colitis model mice, and CsA mitigated this reduction. Compared with CsA, CsA NPs significantly increased occludin protein expression (Fig. 8B). Compared with CsA NPs, LM-CsA NPs significantly increased the protein expression of occludin in colonic tissues (Fig. 8E). In summary, decreases in E-cadherin, ZO-1, and occludin protein levels were detected in chronic colitis and acute colitis model mice, and compared with uncoated CsA NPs, LM-CsA NPs promoted intestinal barrier repair and increased intestinal barrier protein expression.

### Preliminary biosafety assessment

To assess the biosafety of cell membrane-coated CsA NPs, the main organs (such as the heart, liver, spleen, lung, kidney, and testis) of mice with acute and chronic colitis were collected and stained with H&E. The serum levels of ALT, AST, and Cr were also measured. As shown in Figs. S12A and S13A, H&E staining of major organs in the treatment groups was similar to that in the healthy group, and no inflammation or damage was found in these organs. The levels of ALT, AST, and Cr in each treatment group were not significantly different from those in the healthy group (Figs. S12B to D and S13B to D), indicating that the CsA nanoformulations had no obvious side effects on liver or kidney function.

## Discussion

At present, the main goal for IBD treatment in clinical practice is to control the symptoms. Aminosalicylic acid drugs, glucocorticoids, immunomodulators, and other biologics are used for the clinical management of IBD [8]. However, the in vivo adverse effects and instability of these therapeutics cannot be ignored. Nanodrug delivery systems are one approach to overcome this problem. Herein, MMs and LMs were used to coat nanotherapeutics to enhance their ability to target inflammatory sites, since integrin α4β1 and α4β7 on MMs and LMs have strong affinities for CAM in inflammatory tissues [28–30]. Compared to those of CsA NPs, the sizes of the MM-CsA NPs and LM-CsA NPs were slightly greater (Table), because of the cell membrane phospholipid bilayer. The zeta potentials of the MM-CsA NPs and LM-CsA NPs decreased slightly (Table) because both MMs and LMs displayed lower zeta potentials than the CsA NPs. TEM images revealed an outer membrane in the MM-CsA NPs and LM-CsA NPs (Fig. 2Bb and c). CLSM revealed that both the fluorescence probe-labeled membrane and CsA NPs were both present in the nuclei of the RAW 264.7 cells (Fig. 2D and E). These results demonstrated that CsA NPs were successfully coated with MMs and LMs. In addition, the drug release profiles of MM-CsA NPs and LM-CsA NPs were similar to those of CsA NPs (Fig. 2F), suggesting that the cell membrane coating did not obviously alter the physicochemical properties.

The main MM and LM proteins were also found in the MM-CsA NPs and LM-CsA NPs (Fig. S1), suggesting that the coating procedure did not obviously change the protein composition of the cell membrane. Integrin β1 and β7 were expressed both in the cell membranes and in the membrane-coated CsA NPs (Fig. 2C), confirming that the ability of MM-CsA NPs and LM-CsA NPs to target inflammatory sites was maintained. Cellular uptake experiments indicated that MM and LM coatings can reduce the phagocytosis of CsA NPs by normal macrophages (Figs. S4 and S5). Interestingly, the phagocytosis of cell membrane-coated CsA NPs by LPS-activated macrophages or epithelial cells was obviously increased (Figs. S4 to S6). These results implied that cell membrane coating can decrease the phagocytosis of NPs by normal phagocytes and increase the phagocytosis of NPs by inflammatory cells, which may help NPs avoid elimination by the RES and enhance their ability to target inflammatory sites. Furthermore, the special targeting ability of the cell membrane may cause more MM-CsA NPs and LM-CsA NPs to accumulate in the colonic tissues of mice with colitis than uncoated CsA NPs (Fig. 3).

LM-CsA NPs displayed greater therapeutic efficacy in the acute colitis mouse model than the other formulations (Fig. 4). Compared to MM-CsA NPs, LM-coated CsA NPs can effectively prevent elimination by the RES and can be internalized by activated inflammatory cells, which leads to more LM-CsA NPs accumulating in inflammatory tissues. TNF-α and IL-6 are important cytokines related to inflammation. LM-CsA NPs exerted anti-inflammatory effects by significantly decreasing TNF-α and IL-6 expression (Fig. 5A to C), which is in accordance with the in vivo therapeutic results. Moreover, the neutrophils that accumulate at the site of inflammation release MPO, and LM-CsA NPs obviously decreased MPO levels (Fig. 5D), indicating that inflammation in the colon was alleviated by LM-CsA NPs. In addition, ZO-1, E-cadherin, and occludin play important roles in the maintenance of the intestinal epithelial barrier and are dysfunctional in UC. Interestingly, LM-CsA NPs significantly increased ZO-1, E-cadherin, and occludin protein levels (Fig. 5H and I). This increase in protein expression may be a result of LM-CsA NPs significantly relieving colitis symptoms colitis mice, which may increase the expression of these proteins, thereby restoring the function of the intestinal epithelial barrier.

In chronic colitis model mice, the body weights of the mice exhibited periodic changes after treatment due to the reoccurring nature of IBD (Fig. 6C). The in vivo therapeutic efficacy of cell membrane-coated CsA NPs in chronic colitis model mice was similar to that in acute colitis model mice. Taken together, these findings demonstrated that LM-CsA NPs displayed greater therapeutic efficacy in chronic colitis model mice than MM-CsA NPs and CsA NPs because LM-CsA NPs have a strong affinity for inflammatory cells, which is helpful for enhancing the ability of LM-CsA NPs to target inflammatory sites.

In conclusion, inflammation-targeted cell membrane MMs and LMs were used to coat CsA NPs to enhance the targeting ability of nanotherapeutics. TEM and CLSM demonstrated that the CsA NPs were successfully coated with MMs and LMs. The physicochemical properties of the MM-CsA NPs and LM-CsA NPs did not obviously change compared to those of the uncoated CsA NPs. In vitro experiments demonstrated that MM-CsA NPs and LM-CsA NPs, especially LM-CsA NPs, can prevent phagocytosis by normal macrophages and increase internalization by proinflammatory macrophages, which may be helpful for increasing the ability of nanotherapeutics to target inflammatory sites. In vivo biodistribution experiments verified that LM coating can increase the accumulation and prolong the retention time of nanotherapeutics in the colonic tissues of mice with colitis. In vivo therapeutic efficacy confirmed that compared with other treatments, LM-CsA NPs significantly decreased body weight loss, the DAI, the spleen index, the MEICS, and histological score and increased colon length in acute and chronic colitis model mice. LM-CsA NPs exerted anti-inflammatory effects by significantly decreasing TNF-α and IL-6 expression. The protein levels of MPO, ZO-1, E-cadherin, and occludin were decreased in acute and chronic colitis model mice after LM-CsA NP treatment, and intestinal function recovered after treatment. Collectively, LM coating can increase the therapeutic efficacy of CsA NPs for IBD and may be used to enhance the targeting ability of nanotherapeutics for other inflammatory diseases.

## Data Availability

Data sharing not applicable.
