# Supplementary material for: Cell Membrane-Coated Nanotherapeutics for the Targeted Treatment of Acute and Chronic Colitis
Source: Biomater Res. 2024 Nov 7;28:0102. doi: 10.34133/bmr.0102 (PMC11542430; doi:10.34133/bmr.0102)
Supplement: Supplementary 1 — Figs. S1 to S13 Tables S1 to S28 [file bmr.0102.f1.docx]

**Cell membrane-coated nanotherapeutics for the targeted treatment of acute and chronic colitis**

Shan Li^a,b,c^, Lei Chen^c^, Tianyu Wu^c^, Jingfeng Wu^c,^ Hong Yang^c^, Wensheng Chen^c*^, Dinglin Zhang^b*^, Yingxue Hao^d^*.

*a* Army 953 Hospital, Shigatse Branch of Xinqiao Hospital, Army Medical University (Third Military Medical University), Shigatse, Tibet Autonomous Region, 857000, China.

*b* Department of Chemistry, College of Basic Medicine, Army Medical University (Third Military Medical University), Chongqing 400038, China

*c* Department of Gastroenterology, Southwest Hospital, Army Medical University (Third Military Medical University), Chongqing 400038, China

*d* Department of Vascular Surgery, Southwest Hospital, Army Medical University (Third Military Medical University), Chongqing 400038, China

*Corresponding authors:

Yingxue Hao, PhD, Prof.

Department of Vascular Surgery

Southwest Hospital

Army Medical University (Third Military Medical University), Chongqing 400038, China

E-mail: haoyingxue@hotmail.com

Dinglin Zhang, PhD, Prof.

Department of Chemistry

College of Basic Medicine

Army Medical University (Third Military Medical University), Chongqing 400038, China

E-mail: zh18108@163.com, zh18108@tmmu.edu.cn

ORCID: 0000-0003-4400-919X

Wensheng Chen, PhD, Prof.

Department of Gastroenterology

Southwest Hospital

Army Medical University (Third Military Medical University), Chongqing 400038, China

E-mail: wenshengchen@hotmail.com

Table S1, scoring system for calculating the disease activity index (DAI).

|  | Body weight loss | Stool consistency | Bleeding |
| --- | --- | --- | --- |
| 0 | None | Normal | No bleeding |
| 1 | 1–5% | Slightly soft stool | Weak positive hemoccult |
| 2 | 5–10% | Soft stool but still formed | Strong positive hemoccult |
| 3 | 10–15% | Very soft | Visible blood traces in stool |
| 4 | >15% | Watery diarrhea | Rectal bleeding |

Table S2, scoring system for calculating the murine endoscopic index of colitis severity (MEICS).

|  | Thickening of the colon | Changes of the vascular pattern | Fibrin visible | Granularity of the mucosal surface | Stool consistency |
| --- | --- | --- | --- | --- | --- |
| 0 | Transparent | Normal | None | None | Normal and solid |
| 1 | Moderate | Moderate | Little | Moderate | Still shaped |
| 2 | Marked | Marked | Marked | Marked | Unshaped |
| 3 | Non-transparent | Bleeding | Extreme | Extreme | Spread |

Table S3, Scoring system for calculating the histology assessment of colitis.

|  | Inflammation | Crypt Damage | Ulceration | Edema |
| --- | --- | --- | --- | --- |
| 0 | No infiltrate | None | None | Absent |
| 1 | Occasional cell limited to submucosa | Some crypt damage, spaces between crypts | Small, focal ulcers | Present |
| 2 | Significant presence of inflammatory cells in submucosa, limited to focal areas | Larger spaces between crypts, loss of goblet cells, some shortening of crypts | Frequent small ulcers |  |
| 3 | Infiltrate present in both submucosa and lamina propria, limited to focal areas | Large areas without crypts, surrounded by normal crypts | Large areas lacking surface epithelium |  |
| 4 | Large amount of infiltrate in submucosa, lamina propria and surrounding blood vessels, covering large areas of mucosa | No crypts |  |  |
| 5 | Transmural inflammation (mucosa to muscularis) |  |  |  |

Table S4, statistical analysis of ROI analysis in Figure 3B.

| Tukey's multiple comparisons test | Mean Diff. | 95.00% CI of diff. | Below threshold? | Summary | P Value |
| --- | --- | --- | --- | --- | --- |
| 2h | | | | | |
| Cy5 vs. Cy5/CsA NPs | -8.357 | -71.21 to 54.49 | No | ns | 0.9407 |
| Cy5 vs. Cy5/MM-CsA NPs | -41.1 | -107.2 to 24.95 | No | ns | 0.1847 |
| Cy5 vs. Cy5/LM-CsA NPs | -71.12 | -207.5 to 65.26 | No | ns | 0.2086 |
| Cy5/CsA NPs vs. Cy5/MM-CsA NPs | -32.75 | -102.6 to 37.09 | No | ns | 0.3506 |
| Cy5/CsA NPs vs. Cy5/LM-CsA NPs | -62.76 | -192.8 to 67.25 | No | ns | 0.2647 |
| Cy5/MM-CsA NPs vs. Cy5/LM-CsA NPs | -30.02 | -158.5 to 98.51 | No | ns | 0.7045 |
| 6h | | | | | |
| Cy5 vs. Cy5/CsA NPs | 487 | 80.94 to 893.1 | Yes | * | 0.0334 |
| Cy5 vs. Cy5/MM-CsA NPs | 429.3 | 79.93 to 778.7 | Yes | * | 0.0267 |
| Cy5 vs. Cy5/LM-CsA NPs | 479.7 | 56.90 to 902.4 | Yes | * | 0.0383 |
| Cy5/CsA NPs vs. Cy5/MM-CsA NPs | -57.72 | -308.4 to 193.0 | No | ns | 0.6777 |
| Cy5/CsA NPs vs. Cy5/LM-CsA NPs | -7.36 | -112.5 to 97.77 | No | ns | 0.9899 |
| Cy5/MM-CsA NPs vs. Cy5/LM-CsA NPs | 50.36 | -214.4 to 315.1 | No | ns | 0.7346 |
| 12h | | | | | |
| Cy5 vs. Cy5/CsA NPs | 892 | 447.1 to 1337 | Yes | * | 0.0124 |
| Cy5 vs. Cy5/MM-CsA NPs | 599.5 | -119.5 to 1318 | No | ns | 0.0795 |
| Cy5 vs. Cy5/LM-CsA NPs | 857 | 437.0 to 1277 | Yes | * | 0.0108 |
| Cy5/CsA NPs vs. Cy5/MM-CsA NPs | -292.5 | -1197 to 612.1 | No | ns | 0.3522 |
| Cy5/CsA NPs vs. Cy5/LM-CsA NPs | -35.05 | -119.9 to 49.78 | No | ns | 0.3629 |
| Cy5/MM-CsA NPs vs. Cy5/LM-CsA NPs | 257.5 | -632.1 to 1147 | No | ns | 0.4172 |
| 24h | | | | | |
| Cy5 vs. Cy5/CsA NPs | 3.28 | -2.578 to 9.138 | No | ns | 0.1699 |
| Cy5 vs. Cy5/MM-CsA NPs | 0.1513 | -8.544 to 8.846 | No | ns | 0.9998 |
| Cy5 vs. Cy5/LM-CsA NPs | 0.75 | -6.541 to 8.041 | No | ns | 0.9672 |
| Cy5/CsA NPs vs. Cy5/MM-CsA NPs | -3.129 | -13.81 to 7.557 | No | ns | 0.4302 |
| Cy5/CsA NPs vs. Cy5/LM-CsA NPs | -2.53 | -11.38 to 6.325 | No | ns | 0.4558 |
| Cy5/MM-CsA NPs vs. Cy5/LM-CsA NPs | 0.5987 | -8.242 to 9.439 | No | ns | 0.9912 |
| 48h | | | | | |
| Cy5 vs. Cy5/CsA NPs | -0.8017 | -1.581 to -0.02252 | Yes | * | 0.0462 |
| Cy5 vs. Cy5/MM-CsA NPs | -2.043 | -3.328 to -0.7582 | Yes | * | 0.0126 |
| Cy5 vs. Cy5/LM-CsA NPs | -2.715 | -4.291 to -1.138 | Yes | * | 0.0113 |
| Cy5/CsA NPs vs. Cy5/MM-CsA NPs | -1.241 | -2.680 to 0.1973 | No | ns | 0.0697 |
| Cy5/CsA NPs vs. Cy5/LM-CsA NPs | -1.913 | -3.692 to -0.1342 | Yes | * | 0.0426 |
| Cy5/MM-CsA NPs vs. Cy5/LM-CsA NPs | -0.6717 | -2.229 to 0.8857 | No | ns | 0.3987 |
| 72h | | | | | |
| Cy5 vs. Cy5/CsA NPs | -0.5951 | -4.173 to 2.983 | No | ns | 0.7653 |
| Cy5 vs. Cy5/MM-CsA NPs | -2.705 | -6.266 to 0.8549 | No | ns | 0.0894 |
| Cy5 vs. Cy5/LM-CsA NPs | -4.45 | -7.595 to -1.305 | Yes | * | 0.0155 |
| Cy5/CsA NPs vs. Cy5/MM-CsA NPs | -2.11 | -3.016 to -1.205 | Yes | ** | 0.0024 |
| Cy5/CsA NPs vs. Cy5/LM-CsA NPs | -3.855 | -6.960 to -0.7497 | Yes | * | 0.0308 |
| Cy5/MM-CsA NPs vs. Cy5/LM-CsA NPs | -1.745 | -4.833 to 1.343 | No | ns | 0.1655 |

ns, no significance; *, significantly different at p < 0.05

Table S5, statistical analysis of body weight in Figure 4B.

| Time (days) | Groups | | | | | |
| --- | --- | --- | --- | --- | --- | --- |
|  | Healthy | DSS | CsA | CsA NPs | MM-CsA NPs | LM-CsA NPs |
| 1 | - | - | - | - | - | - |
| 2 | - | - | - | - | - | - |
| 3 | - | - | - | - | - | - |
| 4 | - | - | - | *p<0.05 | - | - |
| 5 | - | *p<0.05 | - | *p<0.05 | - | - |
| 6 | - | **p<0.01 | *p<0.05 | *p<0.05 | *p<0.05 | - |
| 7 | - | **p<0.01 | **p<0.01 | **p<0.01 | **p<0.01 | ***p<0.001- |
| 8 | - | ***p<0.001 | ***p<0.001 | **p<0.01 | **p<0.01  #p<0.05 | ***p<0.001 |
| 9 | - | ****p<0.0001 | **p<0.01 | **p<0.01 | *p<0.05  ###p<0.001 | **p<0.01  ##p<0.01 |
| 10 | - | ****p<0.0001 | **p<0.01 | **p<0.01  #p<0.05 | **p<0.01  #p<0.05 | ##p<0.01 |

*p<0.05, **p<0.01, ***p<0.001 and ****p<0.0001 versus the Healthy group

#p<0.05, ##p<0.01 and ###p<0.001 versus DSS group

Table S6, statistical analysis of DAI in Figure 4C.

| Time (days) | Groups | | | | | |
| --- | --- | --- | --- | --- | --- | --- |
|  | Healthy | DSS | CsA | CsA NPs | MM-CsA NPs | LM-CsA NPs |
| 1 | - | - | - | - | - | - |
| 2 | - | - | - | - | - | - |
| 3 | - | *p<0.05 | *p<0.05 | - | - | **p<0.01 |
| 4 | - | **p<0.01 | *p<0.05 | **p<0.01 | - | *p<0.05 |
| 5 | - | ***p<0.001 | **p<0.01 | **p<0.01 | **p<0.01 | *p<0.05  #p<0.05 |
| 6 | - | **p<0.01 | **p<0.01 | *p<0.05 | *p<0.05 | *p<0.05 |
| 7 | - | ***p<0.001 | **p<0.01 | *p<0.05 | *p<0.05 | **p<0.01  #p<0.05 |
| 8 | - | ****p<0.0001 | ***p<0.001 | **p<0.01 | *p<0.05 | *p<0.05  #p<0.05 |
| 9 | - | ****p<0.0001 | ***p<0.001  ##p<0.01 | **p<0.01  ##p<0.01 | *p<0.05  #p<0.05 | ##p<0.01 |
| 10 | - | ****p<0.0001 | **p<0.05  ##p<0.01 | *p<0.05  ##p<0.01 | *p<0.05  ###p<0.001 | ####p<0.0001  +p<0.05 |

*p<0.05, **p<0.01, ***p<0.001 and ****p<0.0001 versus the Healthy group

#p<0.05, ##p<0.01, ###p<0.001 and ####p<0.0001 versus DSS group

+p<0.05 versus CsA group

Table S7, statistical analysis of colon length in Figure 4E

| Tukey's multiple comparisons test | Mean Diff. | 95.00% CI of diff. | Below threshold? | Summary | P Value |
| --- | --- | --- | --- | --- | --- |
| Healthy vs. DSS | 2.072 | 1.301 to 2.842 | Yes | **** | <0.0001 |
| Healthy vs. CsA | 1.282 | 0.5111 to 2.052 | Yes | *** | 0.0003 |
| Healthy vs. CsA NPs | 1.273 | 0.5023 to 2.043 | Yes | *** | 0.0003 |
| Healthy vs. MM-CsA NPs | 0.8037 | 0.03310 to 1.574 | Yes | * | 0.037 |
| Healthy vs. LM-CsA NPs | 0.3143 | -0.4562 to 1.085 | No | ns | 0.8134 |
| DSS vs. CsA | -0.7902 | -1.561 to -0.01960 | Yes | * | 0.0419 |
| DSS vs. CsA NPs | -0.799 | -1.570 to -0.02843 | Yes | * | 0.0386 |
| DSS vs. MM-CsA NPs | -1.268 | -2.039 to -0.4976 | Yes | *** | 0.0003 |
| DSS vs. LM-CsA NPs | -1.758 | -2.528 to -0.9869 | Yes | **** | <0.0001 |
| CsA vs. CsA NPs | -0.00883 | -0.7794 to 0.7617 | No | ns | >0.9999 |
| CsA vs. MM-CsA NPs | -0.478 | -1.249 to 0.2926 | No | ns | 0.4293 |
| CsA vs. LM-CsA NPs | -0.9673 | -1.738 to -0.1968 | Yes | ** | 0.0075 |
| CsA NPs vs. MM-CsA NPs | -0.4692 | -1.240 to 0.3014 | No | ns | 0.4498 |
| CsA NPs vs. LM-CsA NPs | -0.9585 | -1.729 to -0.1879 | Yes | ** | 0.0082 |
| MM-CsA NPs vs. LM-CsA NPs | -0.4893 | -1.260 to 0.2812 | No | ns | 0.4037 |

ns, no significance; *, significantly different at p < 0.05; **, significantly different at p < 0.01; ****significantly different at p < 0.0001.

Table S8, statistical analysis of spleen index in Figure 4F

| Tukey's multiple comparisons test | Mean Diff. | 95.00% CI of diff. | Below threshold? | Summary | P Value |
| --- | --- | --- | --- | --- | --- |
| Healthy vs. DSS | -0.00304 | -0.003867 to -0.002220 | Yes | **** | <0.0001 |
| Healthy vs. CsA | -0.00208 | -0.002898 to -0.001251 | Yes | **** | <0.0001 |
| Healthy vs. CsA NPs | -0.00202 | -0.002839 to -0.001193 | Yes | **** | <0.0001 |
| Healthy vs. MM-CsA NPs | -0.00187 | -0.002691 to -0.001045 | Yes | **** | <0.0001 |
| Healthy vs. LM-CsA NPs | -0.00105 | -0.001874 to -0.0002270 | Yes | ** | 0.0071 |
| DSS vs. CsA | 0.000969 | 0.0001457 to 0.001792 | Yes | * | 0.0146 |
| DSS vs. CsA NPs | 0.001028 | 0.0002044 to 0.001851 | Yes | ** | 0.0087 |
| DSS vs. MM-CsA NPs | 0.001176 | 0.0003524 to 0.001999 | Yes | ** | 0.0023 |
| DSS vs. LM-CsA NPs | 0.001993 | 0.001170 to 0.002817 | Yes | **** | <0.0001 |
| CsA vs. CsA NPs | 5.87E-05 | -0.0007647 to 0.0008821 | No | ns | >0.9999 |
| CsA vs. MM-CsA NPs | 0.000207 | -0.0006167 to 0.001030 | No | ns | 0.969 |
| CsA vs. LM-CsA NPs | 0.001024 | 0.0002009 to 0.001848 | Yes | ** | 0.0089 |
| CsA NPs vs. MM-CsA NPs | 0.000148 | -0.0006754 to 0.0009714 | No | ns | 0.993 |
| CsA NPs vs. LM-CsA NPs | 0.000966 | 0.0001422 to 0.001789 | Yes | * | 0.015 |
| MM-CsA NPs vs. LM-CsA NPs | 0.000818 | -5.794e-006 to 0.001641 | No | ns | 0.0524 |

ns, no significance; *, significantly different at p < 0.05; **, significantly different at p < 0.01; ****significantly different at p < 0.0001.

Table S9, statistical analysis of MEICS in Figure 4G

| Tukey's multiple comparisons test | Mean Diff. | 95.00% CI of diff. | Below threshold? | Summary | P Value |
| --- | --- | --- | --- | --- | --- |
| Health vs. DSS | -13 | -14.82 to -11.18 | Yes | **** | <0.0001 |
| Health vs. CsA | -9.5 | -11.32 to -7.684 | Yes | **** | <0.0001 |
| Health vs. CsA NPs | -7 | -8.816 to -5.184 | Yes | **** | <0.0001 |
| Health vs. MM-CsA NPs | -4.25 | -6.066 to -2.434 | Yes | **** | <0.0001 |
| Health vs. LM-CsA NPs | -3 | -4.816 to -1.184 | Yes | *** | 0.0007 |
| DSS vs. CsA | 3.5 | 1.684 to 5.316 | Yes | *** | 0.0001 |
| DSS vs. CsA NPs | 6 | 4.184 to 7.816 | Yes | **** | <0.0001 |
| DSS vs. MM-CsA NPs | 8.75 | 6.934 to 10.57 | Yes | **** | <0.0001 |
| DSS vs. LM-CsA NPs | 10 | 8.184 to 11.82 | Yes | **** | <0.0001 |
| CsA vs. CsA NPs | 2.5 | 0.6844 to 4.316 | Yes | ** | 0.0042 |
| CsA vs. MM-CsA NPs | 5.25 | 3.434 to 7.066 | Yes | **** | <0.0001 |
| CsA vs. LM-CsA NPs | 6.5 | 4.684 to 8.316 | Yes | **** | <0.0001 |
| CsA NPs vs. MM-CsA NPs | 2.75 | 0.9344 to 4.566 | Yes | ** | 0.0016 |
| CsA NPs vs. LM-CsA NPs | 4 | 2.184 to 5.816 | Yes | **** | <0.0001 |
| MM-CsA NPs vs. LM-CsA NPs | 1.25 | -0.5656 to 3.066 | No | ns | 0.2902 |

ns, no significance; **, significantly different at p < 0.01; ***significantly different at p < 0.001; ****significantly different at p < 0.0001.

Table S10, statistical analysis of histological score in Figure 4H

| Tukey's multiple comparisons test | Mean Diff. | 95.00% CI of diff. | Below threshold? | Summary | P Value |
| --- | --- | --- | --- | --- | --- |
| Health vs. DSS | -11.33 | -13.14 to -9.529 | Yes | **** | <0.0001 |
| Health vs. CsA | -8.833 | -10.64 to -7.029 | Yes | **** | <0.0001 |
| Health vs. CsA NPs | -6.833 | -8.638 to -5.029 | Yes | **** | <0.0001 |
| Health vs. MM-CsA NPs | -5.667 | -7.471 to -3.862 | Yes | **** | <0.0001 |
| Health vs. LM-CsA NPs | -4.333 | -6.138 to -2.529 | Yes | **** | <0.0001 |
| DSS vs. CsA | 2.5 | 0.6958 to 4.304 | Yes | ** | 0.0026 |
| DSS vs. CsA NPs | 4.5 | 2.696 to 6.304 | Yes | **** | <0.0001 |
| DSS vs. MM-CsA NPs | 5.667 | 3.862 to 7.471 | Yes | **** | <0.0001 |
| DSS vs. LM-CsA NPs | 7 | 5.196 to 8.804 | Yes | **** | <0.0001 |
| CsA vs. CsA NPs | 2 | 0.1958 to 3.804 | Yes | * | 0.023 |
| CsA vs. MM-CsA NPs | 3.167 | 1.362 to 4.971 | Yes | *** | 0.0001 |
| CsA vs. LM-CsA NPs | 4.5 | 2.696 to 6.304 | Yes | **** | <0.0001 |
| CsA NPs vs. MM-CsA NPs | 1.167 | -0.6375 to 2.971 | No | ns | 0.384 |
| CsA NPs vs. LM-CsA NPs | 2.5 | 0.6958 to 4.304 | Yes | ** | 0.0026 |
| MM-CsA NPs vs. LM-CsA NPs | 1.333 | -0.4709 to 3.138 | No | ns | 0.2467 |

ns, no significance; *, significantly different at p < 0.05; **, significantly different at p < 0.01; ***significantly different at p < 0.001; ****significantly different at p < 0.0001.

Table S11, statistical analysis of relative expression of TNF-α in Figure 5B

| Tukey's multiple comparisons test | Mean Diff. | 95.00% CI of diff. | Below threshold? | Summary | P Value |
| --- | --- | --- | --- | --- | --- |
| Healthy vs. DSS | -1.002 | -1.745 to -0.2581 | Yes | * | 0.0128 |
| Healthy vs. CsA | -0.3264 | -1.070 to 0.4170 | No | ns | 0.5518 |
| Healthy vs. CsA NPs | -0.11 | -0.8534 to 0.6335 | No | ns | 0.9881 |
| Healthy vs. MM-CsA NPs | -0.03167 | -0.7751 to 0.7118 | No | ns | >0.9999 |
| Healthy vs. LM-CsA NPs | 0.1074 | -0.6361 to 0.8508 | No | ns | 0.9893 |
| DSS vs. CsA | 0.6751 | -0.06837 to 1.419 | No | ns | 0.0741 |
| DSS vs. CsA NPs | 0.8915 | 0.1481 to 1.635 | Yes | * | 0.0223 |
| DSS vs. MM-CsA NPs | 0.9698 | 0.2264 to 1.713 | Yes | * | 0.015 |
| DSS vs. LM-CsA NPs | 1.109 | 0.3654 to 1.852 | Yes | ** | 0.0077 |
| CsA vs. CsA NPs | 0.2165 | -0.5270 to 0.9599 | No | ns | 0.8414 |
| CsA vs. MM-CsA NPs | 0.2948 | -0.4487 to 1.038 | No | ns | 0.6374 |
| CsA vs. LM-CsA NPs | 0.4338 | -0.3096 to 1.177 | No | ns | 0.3107 |
| CsA NPs vs. MM-CsA NPs | 0.07829 | -0.6652 to 0.8217 | No | ns | 0.9974 |
| CsA NPs vs. LM-CsA NPs | 0.2173 | -0.5261 to 0.9608 | No | ns | 0.8394 |
| MM-CsA NPs vs. LM-CsA NPs | 0.1391 | -0.6044 to 0.8825 | No | ns | 0.968 |

ns, no significance; *, significantly different at p < 0.05; **, significantly different at p < 0.01.

Table S12, statistical analysis of relative expression of IL-6 in Figure 5C

| Tukey's multiple comparisons test | Mean Diff. | 95.00% CI of diff. | Below threshold? | Summary | P Value |
| --- | --- | --- | --- | --- | --- |
| Healthy vs. DSS | -0.4049 | -0.7475 to -0.06235 | Yes | * | 0.0239 |
| Healthy vs. CsA | -0.206 | -0.5486 to 0.1365 | No | ns | 0.2876 |
| Healthy vs. CsA NPs | -0.05756 | -0.4001 to 0.2850 | No | ns | 0.9794 |
| Healthy vs. MM-CsA NPs | -0.03878 | -0.3813 to 0.3038 | No | ns | 0.9964 |
| Healthy vs. LM-CsA NPs | -0.01527 | -0.3578 to 0.3273 | No | ns | >0.9999 |
| DSS vs. CsA | 0.1989 | -0.1437 to 0.5414 | No | ns | 0.3147 |
| DSS vs. CsA NPs | 0.3473 | 0.004783 to 0.6899 | Yes | * | 0.0472 |
| DSS vs. MM-CsA NPs | 0.3661 | 0.02357 to 0.7087 | Yes | * | 0.0376 |
| DSS vs. LM-CsA NPs | 0.3896 | 0.04708 to 0.7322 | Yes | * | 0.0285 |
| CsA vs. CsA NPs | 0.1485 | -0.1941 to 0.4910 | No | ns | 0.5629 |
| CsA vs. MM-CsA NPs | 0.1673 | -0.1753 to 0.5098 | No | ns | 0.4593 |
| CsA vs. LM-CsA NPs | 0.1908 | -0.1518 to 0.5333 | No | ns | 0.3478 |
| CsA NPs vs. MM-CsA NPs | 0.01879 | -0.3238 to 0.3613 | No | ns | 0.9999 |
| CsA NPs vs. LM-CsA NPs | 0.0423 | -0.3003 to 0.3849 | No | ns | 0.9947 |
| MM-CsA NPs vs. LM-CsA NPs | 0.02351 | -0.3191 to 0.3661 | No | ns | 0.9997 |

ns, no significance; *, significantly different at p < 0.05.

Table S13, statistical analysis of MPO level in Figure 5D

| Tukey's multiple comparisons test | Mean Diff. | 95.00% CI of diff. | Below threshold? | Summary | P Value |
| --- | --- | --- | --- | --- | --- |
| Healthy vs. DSS | -0.8014 | -0.9029 to -0.6998 | Yes | **** | <0.0001 |
| Healthy vs. CsA | -0.4892 | -0.5907 to -0.3876 | Yes | **** | <0.0001 |
| Healthy vs. CsA NPs | -0.322 | -0.4236 to -0.2205 | Yes | **** | <0.0001 |
| Healthy vs. MM-CsA NPs | -0.2507 | -0.3523 to -0.1492 | Yes | **** | <0.0001 |
| Healthy vs. LM-CsA NPs | -0.09587 | -0.1974 to 0.005690 | No | ns | 0.0708 |
| DSS vs. CsA | 0.3122 | 0.2106 to 0.4138 | Yes | **** | <0.0001 |
| DSS vs. CsA NPs | 0.4794 | 0.3778 to 0.5809 | Yes | **** | <0.0001 |
| DSS vs. MM-CsA NPs | 0.5506 | 0.4491 to 0.6522 | Yes | **** | <0.0001 |
| DSS vs. LM-CsA NPs | 0.7055 | 0.6039 to 0.8071 | Yes | **** | <0.0001 |
| CsA vs. CsA NPs | 0.1672 | 0.06560 to 0.2687 | Yes | *** | 0.0007 |
| CsA vs. MM-CsA NPs | 0.2384 | 0.1369 to 0.3400 | Yes | **** | <0.0001 |
| CsA vs. LM-CsA NPs | 0.3933 | 0.2918 to 0.4949 | Yes | **** | <0.0001 |
| CsA NPs vs. MM-CsA NPs | 0.07129 | -0.03027 to 0.1728 | No | ns | 0.2719 |
| CsA NPs vs. LM-CsA NPs | 0.2262 | 0.1246 to 0.3277 | Yes | **** | <0.0001 |
| MM-CsA NPs vs. LM-CsA NPs | 0.1549 | 0.05331 to 0.2564 | Yes | ** | 0.0015 |

ns, no significance; **, significantly different at p < 0.01; ***significantly different at p < 0.001; ****significantly different at p < 0.0001.

Table S14, statistical analysis of expression levels of ZO-1 protein in Figure 5G

| Tukey's multiple comparisons test | Mean Diff. | 95.00% CI of diff. | Below threshold? | Summary | P Value |
| --- | --- | --- | --- | --- | --- |
| Healthy vs. DSS | 24.6 | 17.16 to 32.04 | Yes | **** | <0.0001 |
| Healthy vs. CsA | 24.9 | 17.46 to 32.34 | Yes | **** | <0.0001 |
| Healthy vs. CsA NPs | 23.07 | 15.63 to 30.50 | Yes | **** | <0.0001 |
| Healthy vs. MM-CsA NPs | 9.913 | 2.474 to 17.35 | Yes | ** | 0.0056 |
| Healthy vs. LM-CsA NPs | 11.42 | 3.984 to 18.86 | Yes | ** | 0.0014 |
| DSS vs. CsA | 0.3015 | -7.138 to 7.741 | No | ns | >0.9999 |
| DSS vs. CsA NPs | -1.532 | -8.971 to 5.907 | No | ns | 0.9848 |
| DSS vs. MM-CsA NPs | -14.68 | -22.12 to -7.245 | Yes | **** | <0.0001 |
| DSS vs. LM-CsA NPs | -13.17 | -20.61 to -5.736 | Yes | *** | 0.0003 |
| CsA vs. CsA NPs | -1.834 | -9.273 to 5.606 | No | ns | 0.9669 |
| CsA vs. MM-CsA NPs | -14.99 | -22.43 to -7.547 | Yes | **** | <0.0001 |
| CsA vs. LM-CsA NPs | -13.48 | -20.92 to -6.037 | Yes | *** | 0.0002 |
| CsA NPs vs. MM-CsA NPs | -13.15 | -20.59 to -5.713 | Yes | *** | 0.0003 |
| CsA NPs vs. LM-CsA NPs | -11.64 | -19.08 to -4.204 | Yes | ** | 0.0012 |
| MM-CsA NPs vs. LM-CsA NPs | 1.51 | -5.929 to 8.949 | No | ns | 0.9857 |

ns, no significance; **, significantly different at p < 0.01; ***significantly different at p < 0.001; ****significantly different at p < 0.0001.

Table S15, statistical analysis of expression levels of E-cadherin protein in Figure 5H

| Tukey's multiple comparisons test | Mean Diff. | 95.00% CI of diff. | Below threshold? | Summary | P Value |
| --- | --- | --- | --- | --- | --- |
| Healthy vs. DSS | 59.32 | 52.95 to 65.68 | Yes | **** | <0.0001 |
| Healthy vs. CsA | 54.38 | 48.02 to 60.74 | Yes | **** | <0.0001 |
| Healthy vs. CsA NPs | 51.76 | 45.40 to 58.12 | Yes | **** | <0.0001 |
| Healthy vs. MM-CsA NPs | 25.25 | 18.89 to 31.61 | Yes | **** | <0.0001 |
| Healthy vs. LM-CsA NPs | 14.07 | 7.712 to 20.44 | Yes | **** | <0.0001 |
| DSS vs. CsA | -4.936 | -11.30 to 1.427 | No | ns | 0.1864 |
| DSS vs. CsA NPs | -7.556 | -13.92 to -1.193 | Yes | * | 0.0148 |
| DSS vs. MM-CsA NPs | -34.07 | -40.43 to -27.71 | Yes | **** | <0.0001 |
| DSS vs. LM-CsA NPs | -45.24 | -51.60 to -38.88 | Yes | **** | <0.0001 |
| CsA vs. CsA NPs | -2.62 | -8.982 to 3.743 | No | ns | 0.7768 |
| CsA vs. MM-CsA NPs | -29.13 | -35.49 to -22.77 | Yes | **** | <0.0001 |
| CsA vs. LM-CsA NPs | -40.31 | -46.67 to -33.94 | Yes | **** | <0.0001 |
| CsA NPs vs. MM-CsA NPs | -26.51 | -32.88 to -20.15 | Yes | **** | <0.0001 |
| CsA NPs vs. LM-CsA NPs | -37.69 | -44.05 to -31.32 | Yes | **** | <0.0001 |
| MM-CsA NPs vs. LM-CsA NPs | -11.17 | -17.54 to -4.811 | Yes | *** | 0.0003 |

ns, no significance; *, significantly different at p < 0.05; ***significantly different at p < 0.001; ****significantly different at p < 0.0001.

Table S16, statistical analysis of expression levels of occludin protein in Figure 5I

| Tukey's multiple comparisons test | Mean Diff. | 95.00% CI of diff. | Below threshold? | Summary | P Value |
| --- | --- | --- | --- | --- | --- |
| Healthy vs. DSS | 0.02725 | 0.01728 to 0.03722 | Yes | **** | <0.0001 |
| Healthy vs. CsA | 0.02025 | 0.01028 to 0.03022 | Yes | **** | <0.0001 |
| Healthy vs. CsA-NPs | 0.016 | 0.006027 to 0.02597 | Yes | *** | 0.0009 |
| Healthy vs. MM-CsA NPs | 0.00675 | -0.003223 to 0.01672 | No | ns | 0.3066 |
| Healthy vs. LM-CsA NPs | -0.00075 | -0.01072 to 0.009223 | No | ns | 0.9999 |
| DSS vs. CsA | -0.007 | -0.01697 to 0.002973 | No | ns | 0.272 |
| DSS vs. CsA-NPs | -0.01125 | -0.02122 to -0.001277 | Yes | * | 0.0219 |
| DSS vs. MM-CsA NPs | -0.0205 | -0.03047 to -0.01053 | Yes | **** | <0.0001 |
| DSS vs. LM-CsA NPs | -0.028 | -0.03797 to -0.01803 | Yes | **** | <0.0001 |
| CsA vs. CsA-NPs | -0.00425 | -0.01422 to 0.005723 | No | ns | 0.7521 |
| CsA vs. MM-CsA NPs | -0.0135 | -0.02347 to -0.003527 | Yes | ** | 0.0049 |
| CsA vs. LM-CsA NPs | -0.021 | -0.03097 to -0.01103 | Yes | **** | <0.0001 |
| CsA-NPs vs. MM-CsA NPs | -0.00925 | -0.01922 to 0.0007228 | No | ns | 0.0782 |
| CsA-NPs vs. LM-CsA NPs | -0.01675 | -0.02672 to -0.006777 | Yes | *** | 0.0005 |
| MM-CsA NPs vs. LM-CsA NPs | -0.0075 | -0.01747 to 0.002473 | No | ns | 0.2112 |

ns, no significance; *, significantly different at p < 0.05; **, significantly different at p < 0.01; ***significantly different at p < 0.001; ****significantly different at p < 0.0001.

Table S17, statistical analysis of body weight in Figure 6B.

| Time (days) | Groups | | | | | |
| --- | --- | --- | --- | --- | --- | --- |
|  | Healthy | DSS | CsA | CsA NPs | MM-CsA NPs | LM-CsA NPs |
| 1-7 | - | - | - | - | - | - |
| 8 | - | *p<0.05 | - | - | - | - |
| 9 | - | - | *p<0.05 | - | *p<0.05 | - |
| 12 | - | *p<0.05 | **p<0.05 | *p<0.05 | *p<0.05 | *p<0.05 |
| 15 | - | *p<0.05 | - | *p<0.05 | *p<0.05 | *p<0.05 |
| 18 | - | **p<0.01 | - | *p<0.05 | *p<0.05 | *p<0.05 |
| 21 | - | **p<0.01 | - | *p<0.05 | - | - |
| 22 | - | ***p<0.001 | #p<0.05 | *p<0.05 | *p<0.05 | #p<0.05 |
| 23 | - | **p<0.01 | #p<0.05 | - | **p<0.01 | **p<0.01 |
| 24 | - | ***p<0.001 | ##p<0.01 | **p<0.01 | *p<0.05 | #p<0.05 |
| 25 | - | ***p<0.001 | **p<0.01  #p<0.05 | ***p<0.001 | **p<0.01 | **p<0.01  #p<0.05 |
| 26 | - | ***p<0.001 | *p<0.05 | *p<0.05  #p<0.05 | *p<0.05  #p<0.05 | #p<0.05 |
| 27 | - | ***p<0.001 | **p<0.01 | **p<0.01  ###p<0.001 | **p<0.01  #p<0.05 | ##p<0.01 |
| 28 | - | ***p<0.001 | **p<0.01 | **p<0.01  ###p<0.001 | ##p<0.01 | **p<0.01  ##p<0.01 |
| 29 | - | ****p<0.0001 | **p<0.01  #p<0.05 | **p<0.01  ##p<0.01 | *p<0.05  ##p<0.01 | **p<0.01  ##p<0.01 |
| 30 | - | ****p<0.0001 | *p<0.05  #p<0.05 | **p<0.01  ##p<0.01 | ##p<0.01 | **p<0.01  #p<0.05 |
| 31 | - | ****p<0.0001 | *p<0.05  #p<0.05 | *p<0.05  #p<0.05 | #p<0.05 | *p<0.05  #p<0.05 |
| 34 | - | ****p<0.0001 | **p<0.01  #p<0.05 | *p<0.05  #p<0.05 | ##p<0.01 | *p<0.05  ##p<0.01 |
| 37 | - | ***p<0.001 | *p<0.05  ##p<0.01 | #p<0.05 | #p<0.05 | ##p<0.01 |
| 39 | - | ****p<0.0001 | **p<0.01  #p<0.05 | *p<0.05  #p<0.05 | #p<0.05 | *p<0.05  ##p<0.01 |
| 41 | - | ***p<0.001 | *p<0.05 | *p<0.05 | - | *p<0.05  ##p<0.01 |
| 42 | - | *p<0.05 | - | - | - | #p<0.05 |
| 43 | - | **p<0.01 | ##p<0.01 | #p<0.05 | - | ###p<0.001 |
| 44 | - | **p<0.01 | ##p<0.01 | #p<0.05 | #p<0.05 | ###p<0.001 |
| 45 | - | **p<0.01 | *p<0.05  #p<0.05 | #p<0.05 | - | ####p<0.0001 |
| 46 | - | ***p<0.001 | **p<0.01  #p<0.05 | *p<0.05  ##p<0.01 | #p<0.05 | **p<0.01  ###p<0.001 |
| 47 | - | ****p<0.0001 | ***p<0.001  #p<0.05 | ***p<0.001  ##p<0.01 | *p<0.05 | ***p<0.001  ###p<0.001 |
| 48 | - | ****p<0.0001 | ****p<0.0001  ##p<0.01 | ***p<0.001 | *p<0.05 | ***p<0.001  ####p<0.0001 |
| 49 | - | ****p<0.0001 | ##p<0.01 | ***p<0.001 | *p<0.05  #p<0.05 | ****p<0.0001  +p<0.05  ###p<0.001 |
| 51 | - | ****p<0.0001 | ****p<0.0001 | ****p<0.0001  #p<0.05 | *p<0.05 | **p<0.01  ###p<0.001  ++p<0.01  $$p<0.01 |
| 52 | - | ****p<0.0001 | ***p<0.001 | ***p<0.001  #p<0.05 | - | ##p<0.01  +++p<0.001  $$$p<0.001 |

*p<0.05, **p<0.01, ***p<0.001 and ****p<0.0001 versus the Healthy group

#p<0.05, ##p<0.01, ###p<0.001 and ####p<0.0001 versus DSS group

+p<0.05, ++p<0.01 and +++p<0.001 versus CsA group

$$p<0.01 and $$$p<0.001 versus CsA NPs group

Table S18, statistical analysis of DAI in Figure 6B.

| Time (days) | Groups | | | | | |
| --- | --- | --- | --- | --- | --- | --- |
|  | Healthy | DSS | CsA | CsA NPs | MM-CsA NPs | LM-CsA NPs |
| 1-3 | - | - | - | - | - | - |
| 4 | - | *p<0.05 | - | - | - | - |
| 5 | - | *p<0.05 | - | *p<0.05 | - | *p<0.05 |
| 6 | - | **p<0.01 | *p<0.05 | **p<0.01 | - | **p<0.01 |
| 7 | - | *p<0.05 | *p<0.05 | *p<0.05 | *p<0.05 | - |
| 8 | - | *p<0.05 | **p<0.01 | - | - | - |
| 9 | - | - | *p<0.05 | - | - | - |
| 12 | - | - | - | *p<0.05 | - | - |
| 15-24 | - | - | - | - | - | - |
| 25 | - | - | - | *p<0.05 | - | *p<0.05 |
| 26 | - | - | *p<0.05 | *p<0.05 | *p<0.05 | - |
| 27 | - | **p<0.01 | - | - | *p<0.05 | - |
| 28 | - | **p<0.01 | - | - | - | #p<0.05 |
| 29 | - | **p<0.01 | - | - | - | ##p<0.01 |
| 30 | - | ***p<0.001 | *p<0.05 | ##p<0.01 | - | ###p<0.001 |
| 31 | - | **p<0.01 | - | - | - | #p<0.05 |
| 34-43 | - | - | - | - | - | - |
| 44 | - | - | ****p<0.0001 | - | - | - |
| 45 | - | *p<0.05 | *p<0.05 | *p<0.05 | *p<0.05 | - |
| 46 | - | ***p<0.001 | - | *p<0.05 | *p<0.05 | - |
| 47 | - | ***p<0.001 | *p<0.05 | *p<0.05 | *p<0.05 | #p<0.05 |
| 48 | - | **p<0.01 | **p<0.01 | *p<0.05 | *p<0.05 | #p<0.05  +p<0.05 |
| 49 | - | ***p<0.001 | **p<0.01 | *p<0.05 | *p<0.05 | - |
| 51 | - | **p<0.01 | ***p<0.001 | *p<0.05 | - | *p<0.05  #p<0.05  +p<0.05 |
| 52 | - | **p<0.01 | **p<0.01 | **p<0.01 | - | *p<0.05  ###p<0.001  ++p<0.01  $p<0.05 |

*p<0.05, **p<0.01 and ***p<0.001 versus the Healthy group

#p<0.05, ##p<0.01 and ###p<0.001 versus DSS group

+p<0.05 and ++p<0.01 versus CsA group

$p<0.05 versus CsA NPs group

Table S19, statistical analysis of colon length in Figure 6D.

| Tukey's multiple comparisons test | Mean Diff. | 95.00% CI of diff. | Below threshold? | Summary | P Value |
| --- | --- | --- | --- | --- | --- |
| Healthy vs. DSS | 2.061 | 1.411 to 2.710 | Yes | **** | <0.0001 |
| Healthy vs. CsA | 1.586 | 0.9365 to 2.235 | Yes | **** | <0.0001 |
| Healthy vs. CsA NPs | 1.432 | 0.7829 to 2.081 | Yes | **** | <0.0001 |
| Healthy vs. MM-CsA NPs | 1.129 | 0.4797 to 1.778 | Yes | *** | 0.0002 |
| Healthy vs. LM-CsA NPs | 0.749 | 0.09987 to 1.398 | Yes | * | 0.0172 |
| DSS vs. CsA | -0.475 | -1.124 to 0.1741 | No | ns | 0.2477 |
| DSS vs. CsA NPs | -0.6286 | -1.278 to 0.02053 | No | ns | 0.0616 |
| DSS vs. MM-CsA NPs | -0.9318 | -1.581 to -0.2827 | Yes | ** | 0.0021 |
| DSS vs. LM-CsA NPs | -1.312 | -1.961 to -0.6625 | Yes | **** | <0.0001 |
| CsA vs. CsA NPs | -0.1536 | -0.8027 to 0.4955 | No | ns | 0.9759 |
| CsA vs. MM-CsA NPs | -0.4568 | -1.106 to 0.1923 | No | ns | 0.285 |
| CsA vs. LM-CsA NPs | -0.8366 | -1.486 to -0.1875 | Yes | ** | 0.0064 |
| CsA NPs vs. MM-CsA NPs | -0.3032 | -0.9523 to 0.3459 | No | ns | 0.7009 |
| CsA NPs vs. LM-CsA NPs | -0.683 | -1.332 to -0.03387 | Yes | * | 0.0351 |
| MM-CsA NPs vs. LM-CsA NPs | -0.3798 | -1.029 to 0.2693 | No | ns | 0.4789 |

ns, no significance; *, significantly different at p < 0.05; **, significantly different at p < 0.01; ***significantly different at p < 0.001; ****significantly different at p < 0.0001.

Table S20, statistical analysis of colon length in Figure 6E.

| Tukey's multiple comparisons test | Mean Diff. | 95.00% CI of diff. | Below threshold? | Summary | P Value |
| --- | --- | --- | --- | --- | --- |
| Healthy vs. DSS | -0.00637 | -0.007583 to -0.005160 | Yes | **** | <0.0001 |
| Healthy vs. CsA | -0.00497 | -0.006182 to -0.003759 | Yes | **** | <0.0001 |
| Healthy vs. CsA NPs | -0.00498 | -0.006189 to -0.003765 | Yes | **** | <0.0001 |
| Healthy vs. MM-CsA NPs | -0.00335 | -0.004556 to -0.002133 | Yes | **** | <0.0001 |
| Healthy vs. LM-CsA NPs | -0.00258 | -0.003792 to -0.001368 | Yes | **** | <0.0001 |
| DSS vs. CsA | 0.001401 | 0.0001893 to 0.002613 | Yes | * | 0.0169 |
| DSS vs. CsA NPs | 0.001395 | 0.0001829 to 0.002606 | Yes | * | 0.0176 |
| DSS vs. MM-CsA NPs | 0.003027 | 0.001815 to 0.004239 | Yes | **** | <0.0001 |
| DSS vs. LM-CsA NPs | 0.003791 | 0.002579 to 0.005003 | Yes | **** | <0.0001 |
| CsA vs. CsA NPs | -6.400e-006 | -0.001218 to 0.001205 | No | ns | >0.9999 |
| CsA vs. MM-CsA NPs | 0.001626 | 0.0004141 to 0.002838 | Yes | ** | 0.0043 |
| CsA vs. LM-CsA NPs | 0.00239 | 0.001178 to 0.003602 | Yes | **** | <0.0001 |
| CsA NPs vs. MM-CsA NPs | 0.001632 | 0.0004205 to 0.002844 | Yes | ** | 0.0042 |
| CsA NPs vs. LM-CsA NPs | 0.002397 | 0.001185 to 0.003608 | Yes | **** | <0.0001 |
| MM-CsA NPs vs. LM-CsA NPs | 0.000764 | -0.0004473 to 0.001976 | No | ns | 0.3979 |

ns, no significance; *, significantly different at p < 0.05; **, significantly different at p < 0.01; ****significantly different at p < 0.0001.

Table S21, statistical analysis of MEICS in Figure 7D.

| Tukey's multiple comparisons test | Mean Diff. | 95.00% CI of diff. | Below threshold? | Summary | P Value |
| --- | --- | --- | --- | --- | --- |
| Health vs. DSS | -13.8 | -15.21 to -12.39 | Yes | **** | <0.0001 |
| Health vs. CsA | -10.8 | -12.21 to -9.394 | Yes | **** | <0.0001 |
| Health vs. CsA NPs | -9.2 | -10.61 to -7.794 | Yes | **** | <0.0001 |
| Health vs. MM-CsA NPs | -7 | -8.406 to -5.594 | Yes | **** | <0.0001 |
| Health vs. LM-CsA NPs | -6 | -7.406 to -4.594 | Yes | **** | <0.0001 |
| DSS vs. CsA | 3 | 1.594 to 4.406 | Yes | **** | <0.0001 |
| DSS vs. CsA NPs | 4.6 | 3.194 to 6.006 | Yes | **** | <0.0001 |
| DSS vs. MM-CsA NPs | 6.8 | 5.394 to 8.206 | Yes | **** | <0.0001 |
| DSS vs. LM-CsA NPs | 7.8 | 6.394 to 9.206 | Yes | **** | <0.0001 |
| CsA vs. CsA NPs | 1.6 | 0.1944 to 3.006 | Yes | * | 0.0192 |
| CsA vs. MM-CsA NPs | 3.8 | 2.394 to 5.206 | Yes | **** | <0.0001 |
| CsA vs. LM-CsA NPs | 4.8 | 3.394 to 6.206 | Yes | **** | <0.0001 |
| CsA NPs vs. MM-CsA NPs | 2.2 | 0.7944 to 3.606 | Yes | *** | 0.0008 |
| CsA NPs vs. LM-CsA NPs | 3.2 | 1.794 to 4.606 | Yes | **** | <0.0001 |
| MM-CsA NPs vs. LM-CsA NPs | 1 | -0.4056 to 2.406 | No | ns | 0.2743 |

ns, no significance; *, significantly different at p < 0.05; ***significantly different at p < 0.001; ****significantly different at p < 0.0001.

Table S22, statistical analysis of histological score in Figure 7D.

| Tukey's multiple comparisons test | Mean Diff. | 95.00% CI of diff. | Below threshold? | Summary | P Value |
| --- | --- | --- | --- | --- | --- |
| Health vs. DSS | -12 | -14.08 to -9.918 | Yes | **** | <0.0001 |
| Health vs. CsA | -9.333 | -11.42 to -7.251 | Yes | **** | <0.0001 |
| Health vs. CsA NPs | -6.5 | -8.582 to -4.418 | Yes | **** | <0.0001 |
| Health vs. MM-CsA NPs | -6 | -8.082 to -3.918 | Yes | **** | <0.0001 |
| Health vs. LM-CsA NPs | -4.333 | -6.415 to -2.251 | Yes | **** | <0.0001 |
| DSS vs. CsA | 2.667 | 0.5847 to 4.749 | Yes | ** | 0.0061 |
| DSS vs. CsA NPs | 5.5 | 3.418 to 7.582 | Yes | **** | <0.0001 |
| DSS vs. MM-CsA NPs | 6 | 3.918 to 8.082 | Yes | **** | <0.0001 |
| DSS vs. LM-CsA NPs | 7.667 | 5.585 to 9.749 | Yes | **** | <0.0001 |
| CsA vs. CsA NPs | 2.833 | 0.7514 to 4.915 | Yes | ** | 0.0032 |
| CsA vs. MM-CsA NPs | 3.333 | 1.251 to 5.415 | Yes | *** | 0.0004 |
| CsA vs. LM-CsA NPs | 5 | 2.918 to 7.082 | Yes | **** | <0.0001 |
| CsA NPs vs. MM-CsA NPs | 0.5 | -1.582 to 2.582 | No | ns | 0.9765 |
| CsA NPs vs. LM-CsA NPs | 2.167 | 0.08474 to 4.249 | Yes | * | 0.0376 |
| MM-CsA NPs vs. LM-CsA NPs | 1.667 | -0.4153 to 3.749 | No | ns | 0.1766 |

ns, no significance; *, significantly different at p < 0.05; **, significantly different at p < 0.01; ***significantly different at p < 0.001; ****significantly different at p < 0.0001.

Table S23, statistical analysis of relative expression of TNF-α in Figure 7F.

| Tukey's multiple comparisons test | Mean Diff. | 95.00% CI of diff. | Below threshold? | Summary | P Value |
| --- | --- | --- | --- | --- | --- |
| Healthy vs. DSS | -1.007 | -1.908 to -0.1058 | Yes | * | 0.0308 |
| Healthy vs. CsA | -0.5351 | -1.436 to 0.3662 | No | ns | 0.2974 |
| Healthy vs. CsA NPs | -0.2825 | -1.184 to 0.6187 | No | ns | 0.8019 |
| Healthy vs. MM-CsA NPs | -0.2927 | -1.194 to 0.6085 | No | ns | 0.7807 |
| Healthy vs. LM-CsA NPs | 0.005961 | -0.8953 to 0.9072 | No | ns | >0.9999 |
| DSS vs. CsA | 0.472 | -0.4292 to 1.373 | No | ns | 0.3989 |
| DSS vs. CsA NPs | 0.7246 | -0.1767 to 1.626 | No | ns | 0.1172 |
| DSS vs. MM-CsA NPs | 0.7144 | -0.1869 to 1.616 | No | ns | 0.1232 |
| DSS vs. LM-CsA NPs | 1.013 | 0.1118 to 1.914 | Yes | * | 0.03 |
| CsA vs. CsA NPs | 0.2525 | -0.6487 to 1.154 | No | ns | 0.8595 |
| CsA vs. MM-CsA NPs | 0.2423 | -0.6589 to 1.144 | No | ns | 0.8772 |
| CsA vs. LM-CsA NPs | 0.541 | -0.3602 to 1.442 | No | ns | 0.2891 |
| CsA NPs vs. MM-CsA NPs | -0.0102 | -0.9115 to 0.8911 | No | ns | >0.9999 |
| CsA NPs vs. LM-CsA NPs | 0.2885 | -0.6128 to 1.190 | No | ns | 0.7896 |
| MM-CsA NPs vs. LM-CsA NPs | 0.2987 | -0.6026 to 1.200 | No | ns | 0.7681 |

ns, no significance; *, significantly different at p < 0.05.

Table S24, statistical analysis of relative expression of IL-6 in Figure 7G.

| Tukey's multiple comparisons test | Mean Diff. | 95.00% CI of diff. | Below threshold? | Summary | P Value |
| --- | --- | --- | --- | --- | --- |
| Healthy vs. DSS | -0.2092 | -0.3711 to -0.04724 | Yes | * | 0.0157 |
| Healthy vs. CsA | -0.07484 | -0.2368 to 0.08707 | No | ns | 0.5071 |
| Healthy vs. CsA NPs | 0.05145 | -0.1105 to 0.2134 | No | ns | 0.794 |
| Healthy vs. MM-CsA NPs | 0.1256 | -0.03632 to 0.2875 | No | ns | 0.1329 |
| Healthy vs. LM-CsA NPs | 0.1086 | -0.05331 to 0.2705 | No | ns | 0.2122 |
| DSS vs. CsA | 0.1343 | -0.02760 to 0.2962 | No | ns | 0.1046 |
| DSS vs. CsA NPs | 0.2606 | 0.09868 to 0.4225 | Yes | ** | 0.0052 |
| DSS vs. MM-CsA NPs | 0.3347 | 0.1728 to 0.4967 | Yes | ** | 0.0014 |
| DSS vs. LM-CsA NPs | 0.3178 | 0.1558 to 0.4797 | Yes | ** | 0.0018 |
| CsA vs. CsA NPs | 0.1263 | -0.03563 to 0.2882 | No | ns | 0.1304 |
| CsA vs. MM-CsA NPs | 0.2004 | 0.03852 to 0.3623 | Yes | * | 0.0192 |
| CsA vs. LM-CsA NPs | 0.1834 | 0.02153 to 0.3454 | Yes | * | 0.029 |
| CsA NPs vs. MM-CsA NPs | 0.07415 | -0.08777 to 0.2361 | No | ns | 0.5153 |
| CsA NPs vs. LM-CsA NPs | 0.05716 | -0.1048 to 0.2191 | No | ns | 0.7256 |
| MM-CsA NPs vs. LM-CsA NPs | -0.01699 | -0.1789 to 0.1449 | No | ns | 0.9975 |

ns, no significance; *, significantly different at p < 0.05; **, significantly different at p < 0.01.

Table S25, statistical analysis of relative expression of MPO activity in Figure 7H

| Tukey's multiple comparisons test | Mean Diff. | 95.00% CI of diff. | Below threshold? | Summary | P Value |
| --- | --- | --- | --- | --- | --- |
| Healthy vs. DSS | -0.9784 | -1.058 to -0.8992 | Yes | **** | <0.0001 |
| Healthy vs. CsA | -0.4941 | -0.5733 to -0.4149 | Yes | **** | <0.0001 |
| Healthy vs. CsA NPs | -0.3441 | -0.4233 to -0.2649 | Yes | **** | <0.0001 |
| Healthy vs. MM-CsA NPs | -0.1745 | -0.2537 to -0.09533 | Yes | **** | <0.0001 |
| Healthy vs. LM-CsA NPs | -0.05162 | -0.1308 to 0.02758 | No | ns | 0.344 |
| DSS vs. CsA | 0.4843 | 0.4051 to 0.5635 | Yes | **** | <0.0001 |
| DSS vs. CsA NPs | 0.6342 | 0.5550 to 0.7134 | Yes | **** | <0.0001 |
| DSS vs. MM-CsA NPs | 0.8038 | 0.7246 to 0.8830 | Yes | **** | <0.0001 |
| DSS vs. LM-CsA NPs | 0.9267 | 0.8475 to 1.006 | Yes | **** | <0.0001 |
| CsA vs. CsA NPs | 0.15 | 0.07075 to 0.2292 | Yes | *** | 0.0001 |
| CsA vs. MM-CsA NPs | 0.3196 | 0.2404 to 0.3988 | Yes | **** | <0.0001 |
| CsA vs. LM-CsA NPs | 0.4425 | 0.3633 to 0.5217 | Yes | **** | <0.0001 |
| CsA NPs vs. MM-CsA NPs | 0.1696 | 0.09042 to 0.2488 | Yes | **** | <0.0001 |
| CsA NPs vs. LM-CsA NPs | 0.2925 | 0.2133 to 0.3717 | Yes | **** | <0.0001 |
| MM-CsA NPs vs. LM-CsA NPs | 0.1229 | 0.04371 to 0.2021 | Yes | ** | 0.0013 |

ns, no significance; **, significantly different at p < 0.01; ***significantly different at p < 0.001; ****significantly different at p < 0.0001.

Table S26, statistical analysis of expression of ZO-1 protein in Figure 8C

| Tukey's multiple comparisons test | Mean Diff. | 95.00% CI of diff. | Below threshold? | Summary | P Value |
| --- | --- | --- | --- | --- | --- |
| Healthy vs. DSS | 43.8 | 41.74 to 45.86 | Yes | **** | <0.0001 |
| Healthy vs. CsA | 39.03 | 36.97 to 41.08 | Yes | **** | <0.0001 |
| Healthy vs. CsA NPs | 32.57 | 30.52 to 34.63 | Yes | **** | <0.0001 |
| Healthy vs. MM-CsA NPs | 26.16 | 24.11 to 28.22 | Yes | **** | <0.0001 |
| Healthy vs. LM-CsA NPs | 26.16 | 24.11 to 28.22 | Yes | **** | <0.0001 |
| DSS vs. CsA | -4.773 | -6.831 to -2.715 | Yes | **** | <0.0001 |
| DSS vs. CsA NPs | -11.23 | -13.28 to -9.167 | Yes | **** | <0.0001 |
| DSS vs. MM-CsA NPs | -17.64 | -19.69 to -15.58 | Yes | **** | <0.0001 |
| DSS vs. LM-CsA NPs | -17.64 | -19.69 to -15.58 | Yes | **** | <0.0001 |
| CsA vs. CsA NPs | -6.452 | -8.510 to -4.394 | Yes | **** | <0.0001 |
| CsA vs. MM-CsA NPs | -12.86 | -14.92 to -10.80 | Yes | **** | <0.0001 |
| CsA vs. LM-CsA NPs | -12.86 | -14.92 to -10.80 | Yes | **** | <0.0001 |
| CsA NPs vs. MM-CsA NPs | -6.41 | -8.468 to -4.352 | Yes | **** | <0.0001 |
| CsA NPs vs. LM-CsA NPs | -6.41 | -8.468 to -4.352 | Yes | **** | <0.0001 |
| MM-CsA NPs vs. LM-CsA NPs | 0 | -2.058 to 2.058 | No | ns | >0.9999 |

ns, no significance; ****significantly different at p < 0.0001.

Table S27, statistical analysis of expression of E-cadherin protein in Figure 8D

| Tukey's multiple comparisons test | Mean Diff. | 95.00% CI of diff. | Below threshold? | Summary | P Value |
| --- | --- | --- | --- | --- | --- |
| Healthy vs. DSS | 78.57 | 69.45 to 87.69 | Yes | **** | <0.0001 |
| Healthy vs. CsA | 71.82 | 62.70 to 80.93 | Yes | **** | <0.0001 |
| Healthy vs. CsA NPs | 47.71 | 38.59 to 56.83 | Yes | **** | <0.0001 |
| Healthy vs. MM-CsA NPs | 17.73 | 8.617 to 26.85 | Yes | **** | <0.0001 |
| Healthy vs. LM-CsA NPs | -9.072 | -18.19 to 0.04505 | No | ns | 0.0516 |
| DSS vs. CsA | -6.755 | -15.87 to 2.362 | No | ns | 0.2238 |
| DSS vs. CsA NPs | -30.86 | -39.98 to -21.75 | Yes | **** | <0.0001 |
| DSS vs. MM-CsA NPs | -60.84 | -69.95 to -51.72 | Yes | **** | <0.0001 |
| DSS vs. LM-CsA NPs | -87.64 | -96.76 to -78.53 | Yes | **** | <0.0001 |
| CsA vs. CsA NPs | -24.11 | -33.23 to -14.99 | Yes | **** | <0.0001 |
| CsA vs. MM-CsA NPs | -54.08 | -63.20 to -44.97 | Yes | **** | <0.0001 |
| CsA vs. LM-CsA NPs | -80.89 | -90.01 to -71.77 | Yes | **** | <0.0001 |
| CsA NPs vs. MM-CsA NPs | -29.97 | -39.09 to -20.86 | Yes | **** | <0.0001 |
| CsA NPs vs. LM-CsA NPs | -56.78 | -65.90 to -47.66 | Yes | **** | <0.0001 |
| MM-CsA NPs vs. LM-CsA NPs | -26.81 | -35.92 to -17.69 | Yes | **** | <0.0001 |

ns, no significance; ****significantly different at p < 0.0001.

Table S28, statistical analysis of expression of occludin protein in Figure 8E

| Tukey's multiple comparisons test | Mean Diff. | 95.00% CI of diff. | Below threshold? | Summary | P Value |
| --- | --- | --- | --- | --- | --- |
| Healthy vs. DSS | 0.041 | 0.03235 to 0.04965 | Yes | **** | <0.0001 |
| Healthy vs. CsA | 0.02975 | 0.02110 to 0.03840 | Yes | **** | <0.0001 |
| Healthy vs. CsA-NPs | 0.01525 | 0.006595 to 0.02390 | Yes | *** | 0.0003 |
| Healthy vs. MM-CsA NPs | 0.00975 | 0.001095 to 0.01840 | Yes | * | 0.0222 |
| Healthy vs. LM-CsA NPs | 0.00375 | -0.004905 to 0.01240 | No | ns | 0.7396 |
| DSS vs. CsA | -0.01125 | -0.01990 to -0.002595 | Yes | ** | 0.007 |
| DSS vs. CsA-NPs | -0.02575 | -0.03440 to -0.01710 | Yes | **** | <0.0001 |
| DSS vs. MM-CsA NPs | -0.03125 | -0.03990 to -0.02260 | Yes | **** | <0.0001 |
| DSS vs. LM-CsA NPs | -0.03725 | -0.04590 to -0.02860 | Yes | **** | <0.0001 |
| CsA vs. CsA-NPs | -0.0145 | -0.02315 to -0.005845 | Yes | *** | 0.0006 |
| CsA vs. MM-CsA NPs | -0.02 | -0.02865 to -0.01135 | Yes | **** | <0.0001 |
| CsA vs. LM-CsA NPs | -0.026 | -0.03465 to -0.01735 | Yes | **** | <0.0001 |
| CsA-NPs vs. MM-CsA NPs | -0.0055 | -0.01415 to 0.003155 | No | ns | 0.3698 |
| CsA-NPs vs. LM-CsA NPs | -0.0115 | -0.02015 to -0.002845 | Yes | ** | 0.0058 |
| MM-CsA NPs vs. LM-CsA NPs | -0.006 | -0.01465 to 0.002655 | No | ns | 0.2836 |

ns, no significance; **, significantly different at p < 0.01; ***significantly different at p < 0.001; ****significantly different at p < 0.0001.


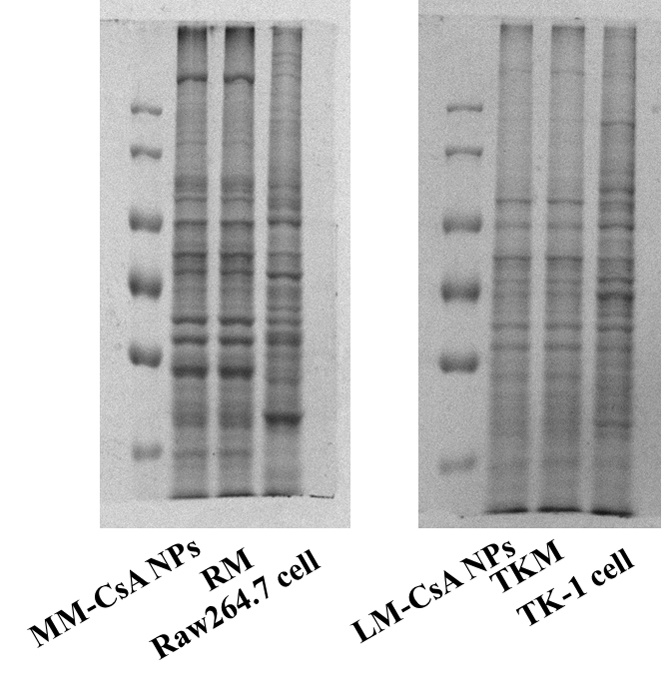


Figure S1, protein profiles of cell membrane-encapsulated NPs, membrane and cell by SDS‒PAGE.


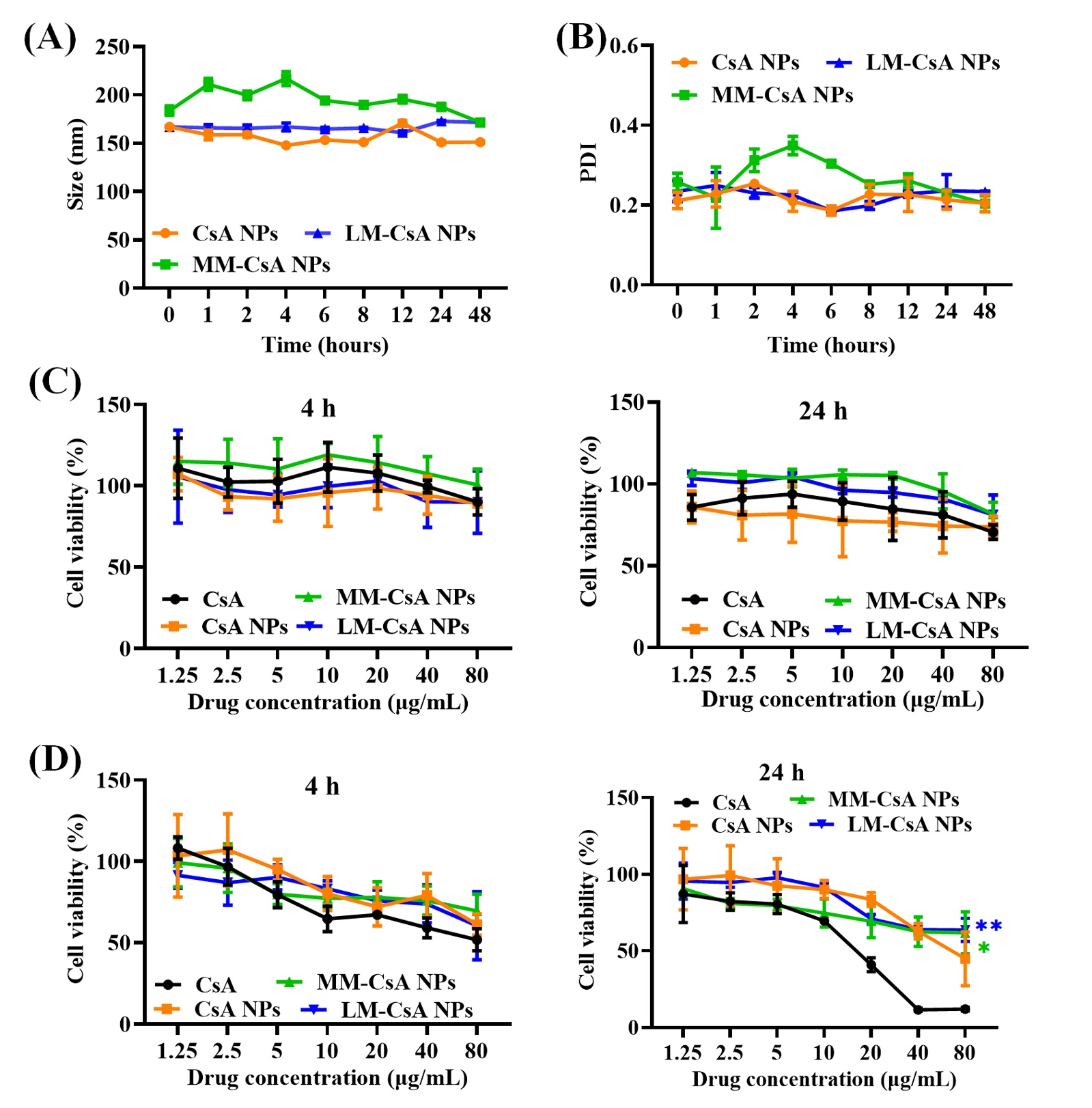


Figure S2, stability and ctotoxicity of NPs. Size (A) and PDI (B) of the CsA NPs, MM-CsA NPs, and LM-CsA NPs in ultrapure water at different time points. (C) CCK-8 assays determined the viability of Mc 38.2 cells treated with various NPs for 4 h and 24 h. (D) CCK-8 assays determined the viability of RAW 264.7 cells treated with various NPs for 4 h and 24 h.


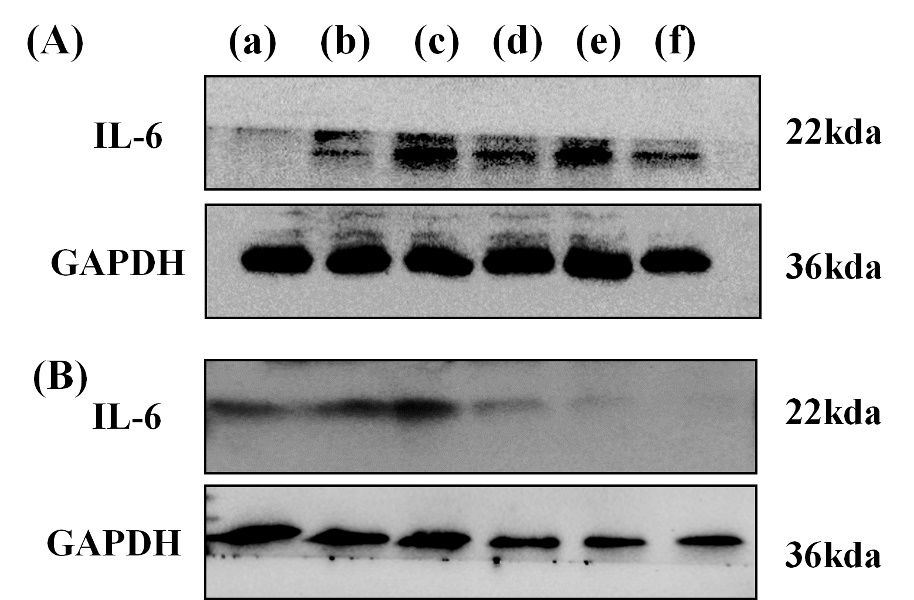


Figure S3, western blot analysis of IL-6 expression in RAW 264.7 (A) cells and HUVECs (B) treated with CsA or its nanoformulations. GAPDH was used as the internal reference protein. (a) control; (b) LPS treatment; (c) LPS + CsA treatment; (d) LPS + CsA NPs treatment; (e) LPS + MM-CsA NPs treatment; (f) LPS + LM-CsA NPs treatment.


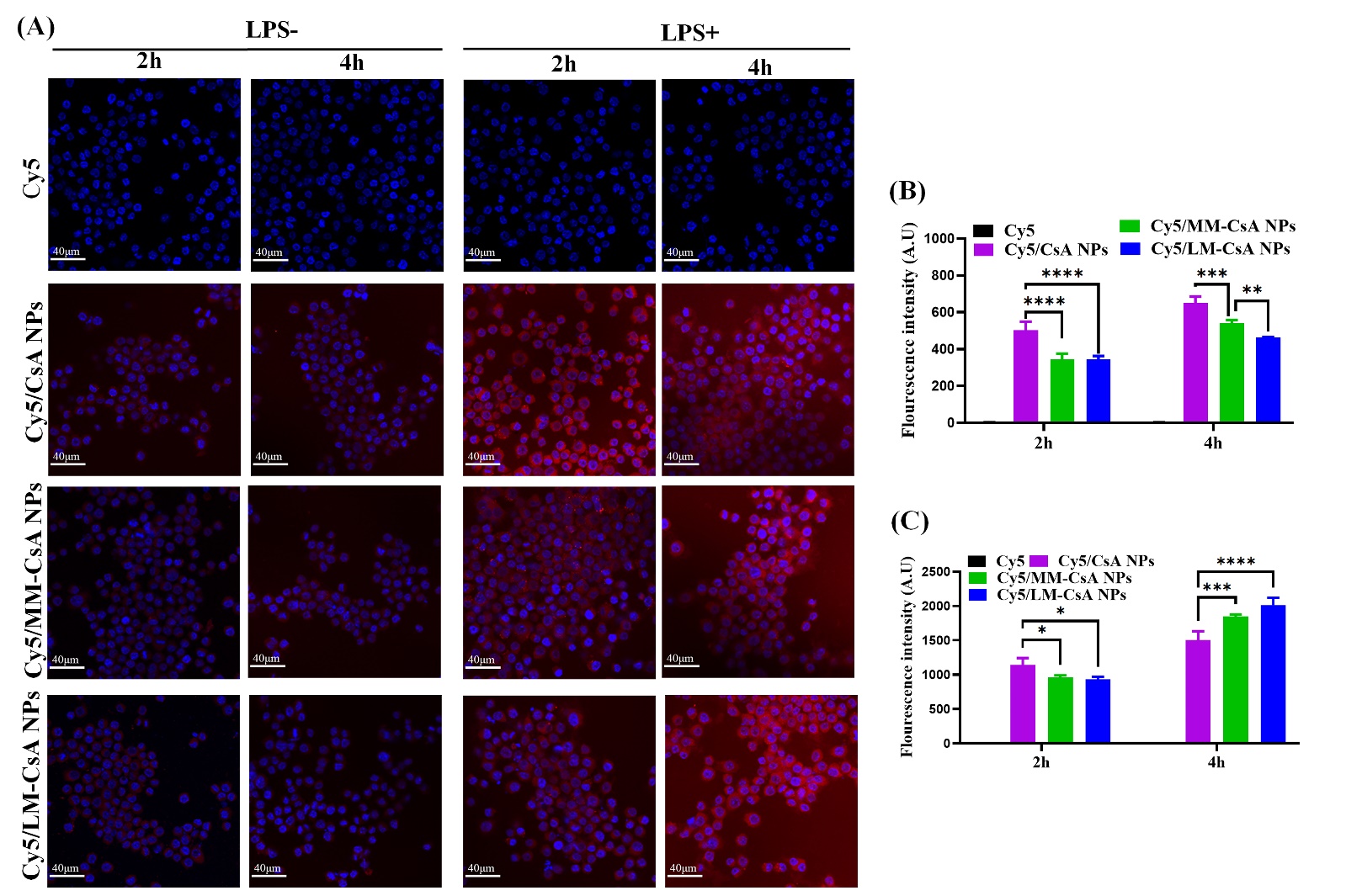


Figure S4, cellular uptake of NPs by RAW 264.7 cells with or without LPS-treatment detected by CLSM. (A) CLSM images of the cellular uptake of Cy5-labeled NPs by RAW 264.7 cell. Red indicates NPs, and blue indicates DAPI. Semiquantitative analysis of Cy5 fluorescence intensity without (B) or with (C) LPS-treatment. *, significantly different at p < 0.05; **, significantly different at p < 0.01; ***significantly different at p < 0.001; ****significantly different at p < 0.0001.


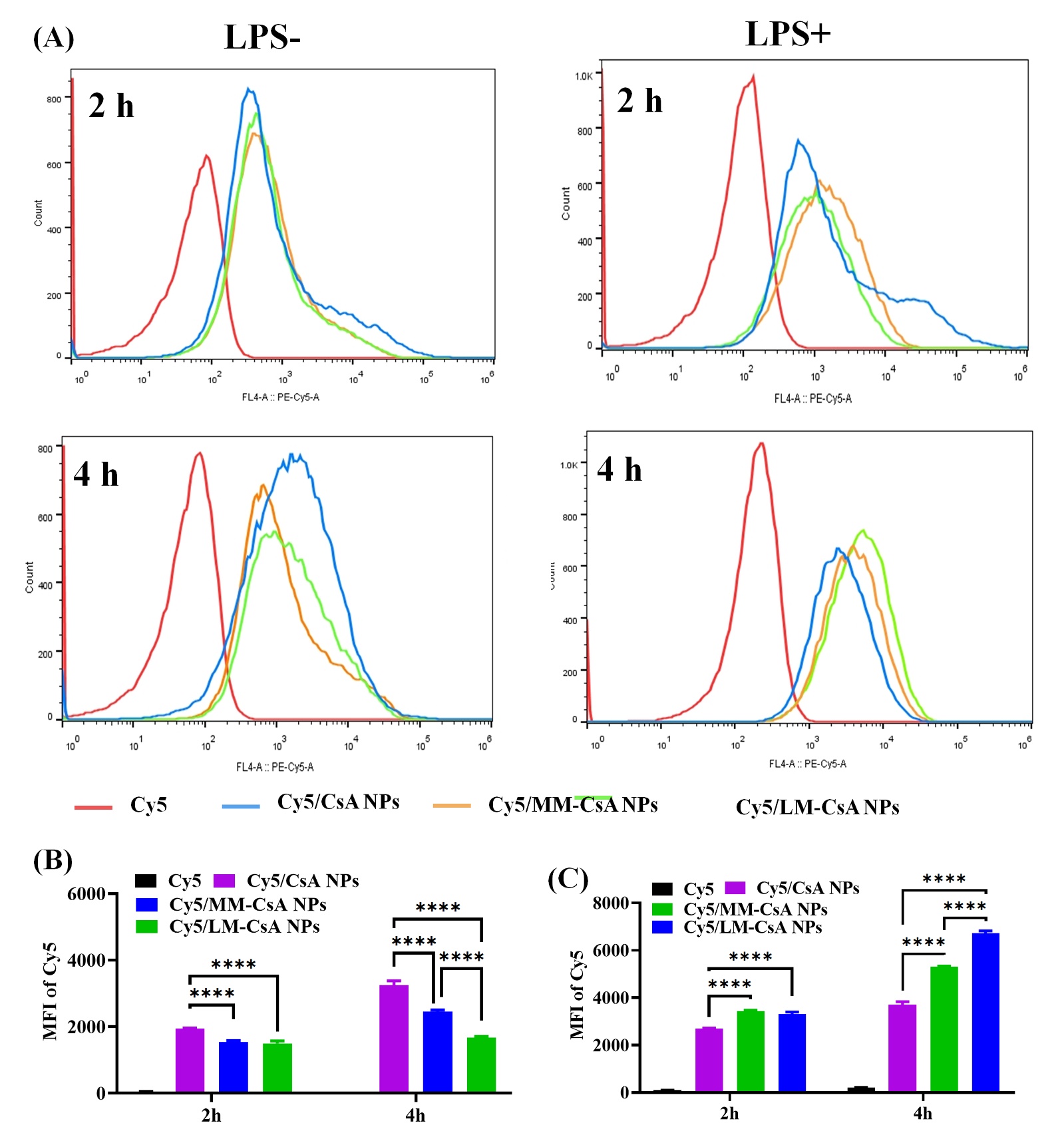


Figure S5, flow cytometry detected cellular uptake of the NPs. (A) FACS analysis on RAW 264.7 cell with or without LPS-treatment at 2 h and 4 h. Quantitative analysis cellular uptake without (B) or with (C) LPS-treatment. ****, significantly different at *p* < 0.0001.


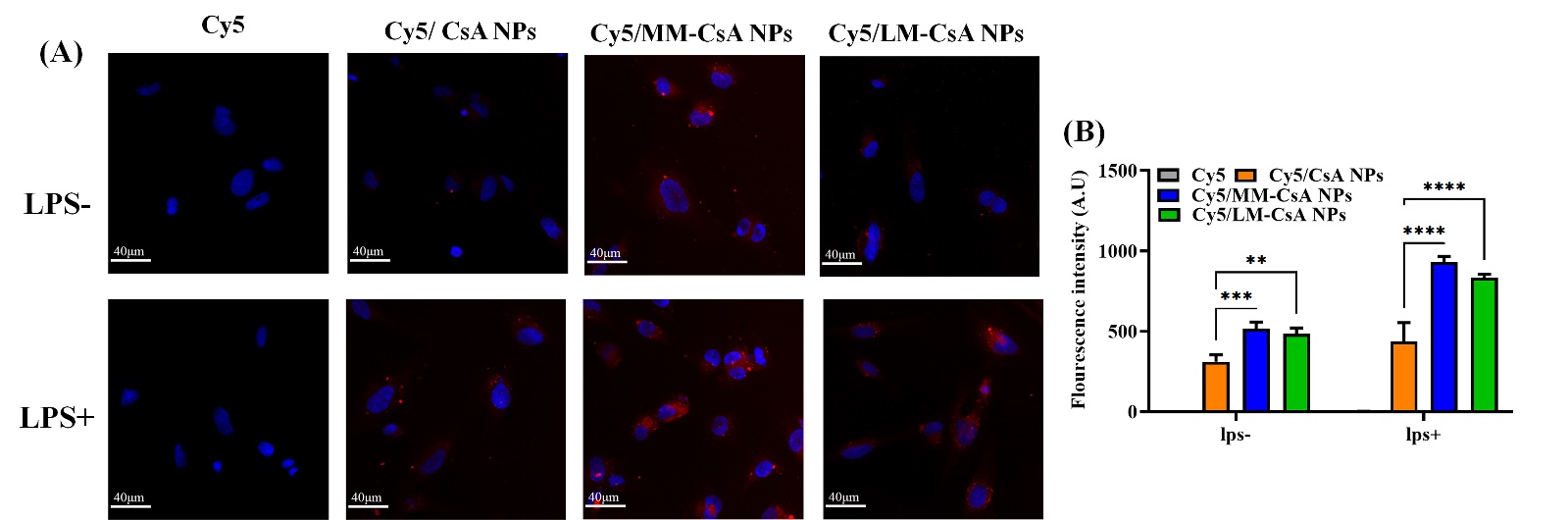


Figure S6, cellular uptake of NPs by HUVECs without or with LPS-treatment detected by CLSM. (A) CLSM images of the cellular uptake of Cy5-labeled NPs by HUVECs. Red indicates NPs, and blue indicates DAPI. (B) Semiquantitative analysis of Cy5 fluorescence intensity. **, significantly different at p < 0.01; ***significantly different at p < 0.001; ****significantly different at p < 0.0001.


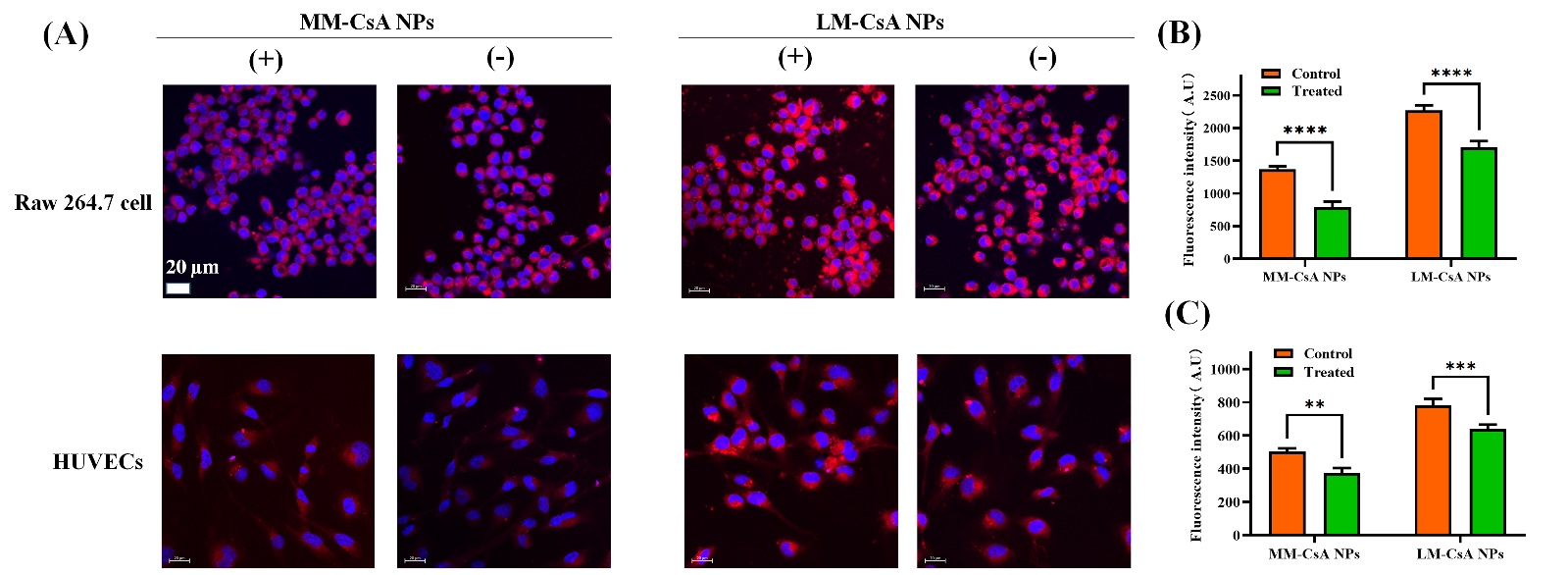


Figure S7, integrin inhibition experiments detected by CLSM. (A) CLSM images of the cellular uptake of Cy5-labeled NPs by RAW 264.7 cell and HUVECs, respectively. Red indicates NPs, blue indicates DAPI, (+) indicates that integrins are not inhibited and (-) indicates that integrins are inhibited. (B) Semiquantitative analysis of Cy5 fluorescence intensity in RAW 264.7 cell. (C) Semiquantitative analysis of Cy5 fluorescence intensity in HUVECs. **, significantly different at p < 0.01; ***, significantly different at p < 0.001; ****, significantly different at p < 0.0001.


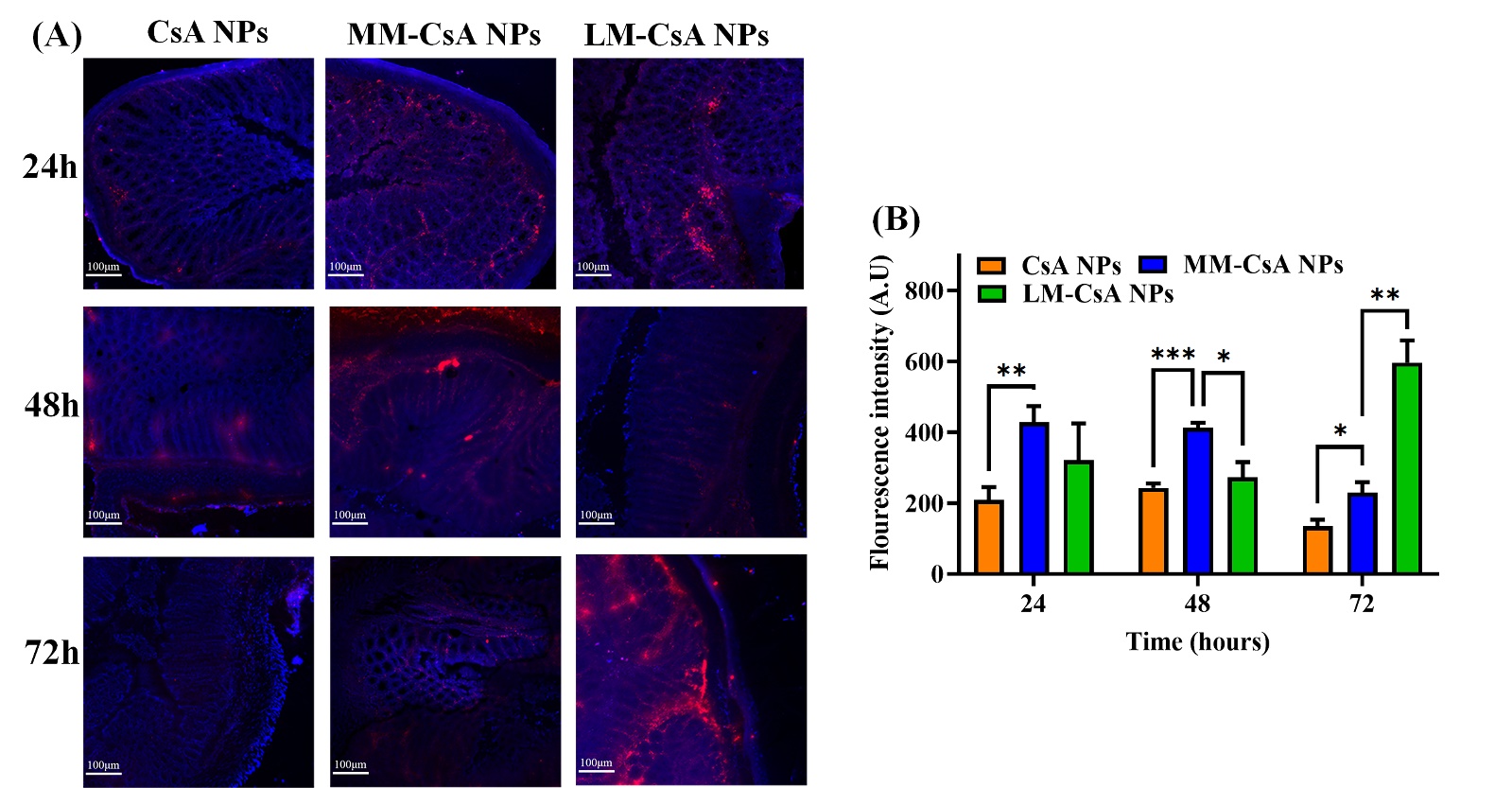


Figure S8, (A) CLSM images of frozen sections of mouse colon tissue. Red indicates NPs, and blue indicates DAPI. (B) Statistical analysis of Cy5 fluorescence intensity in mouse colon. *, significantly different at p < 0.05; **, significantly different at p < 0.01; ***significantly different at p < 0.001.


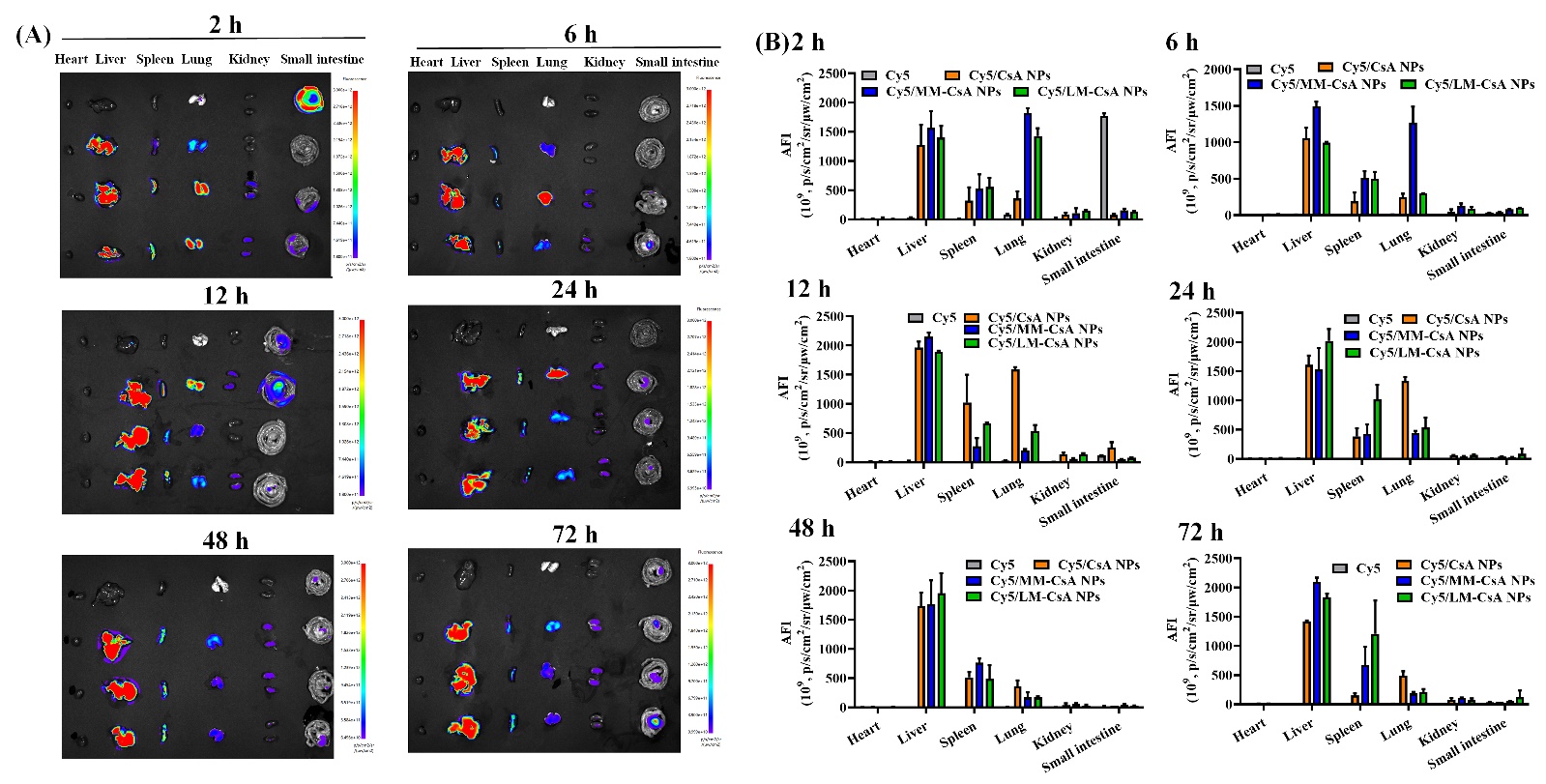


Figure S9, biodistribution of free Cy5 and Cy5/NPs in DSS-induced colitis mice. (A) Fluorescence intensity of primary tissues at different time points; (B) Region of interest (ROI) analysis of fluorescence intensity in primary tissues at different time points.


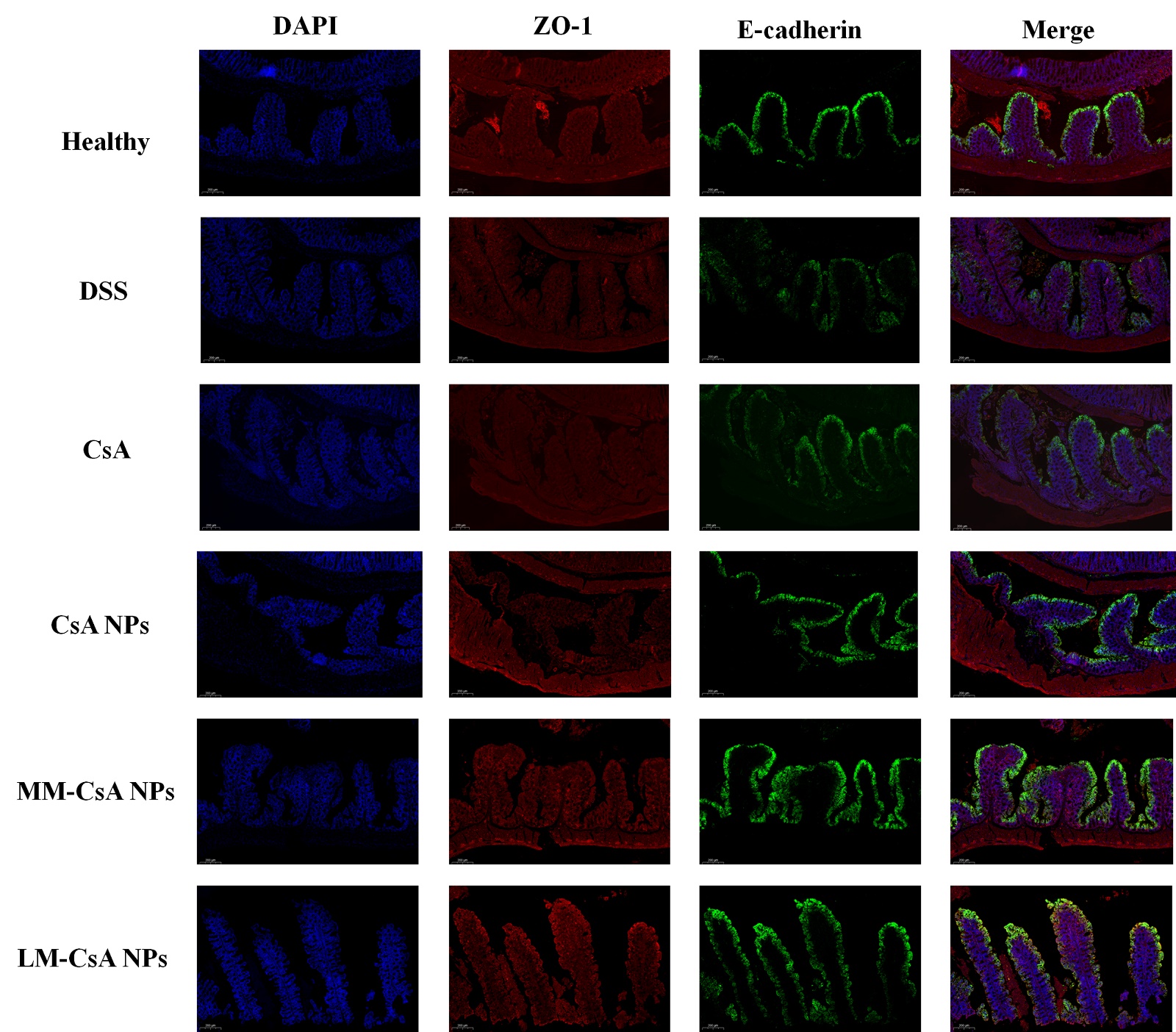


Figure S10, the channel images in Figure 5E.


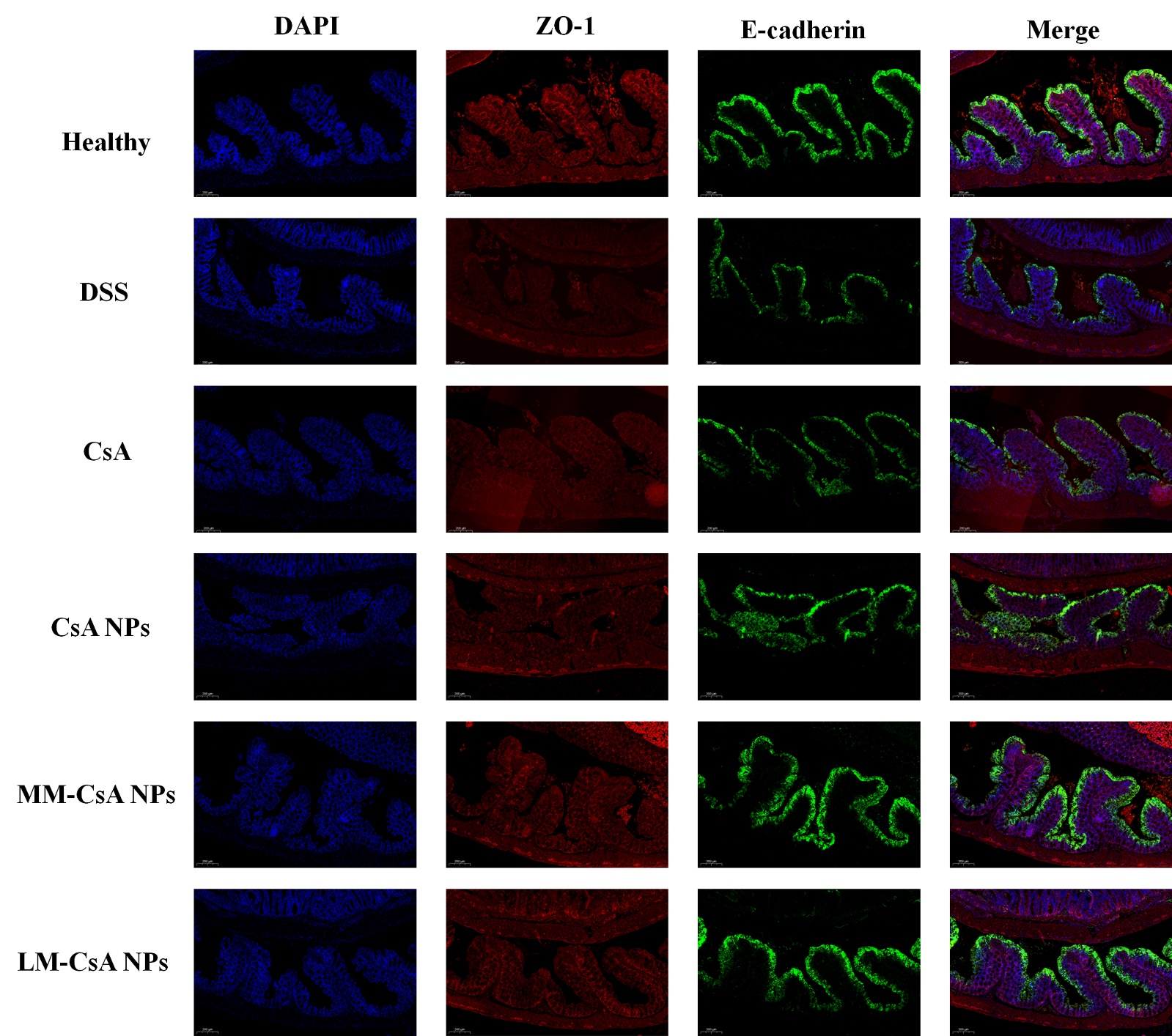


Figure S11, the channel images in Figure 8A.


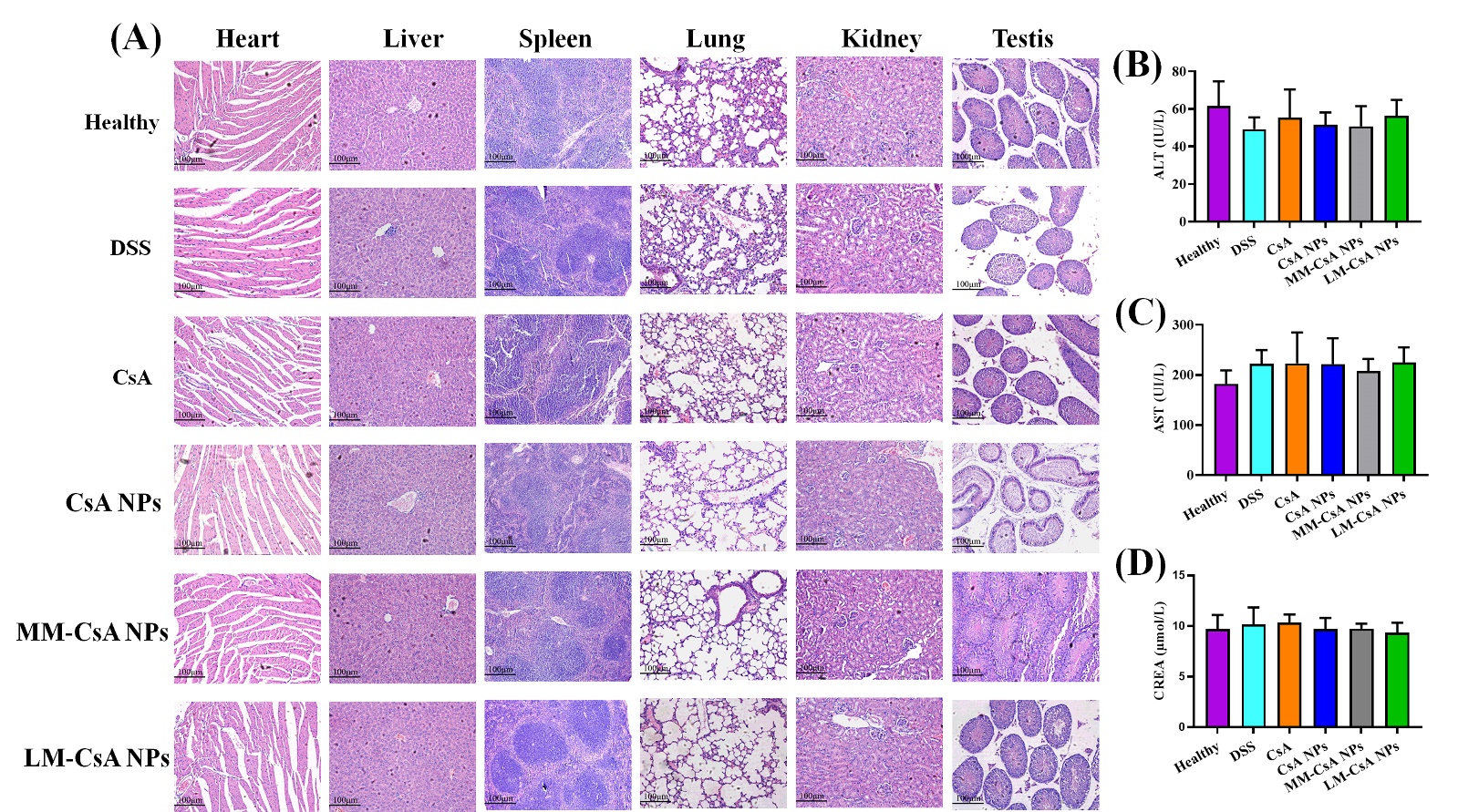


Figure S12, *in vivo* biosafety evaluation of NPs in acute colitis. H&E-staining of main organs slices from mice in each group (A); and level of ALT (B), AST (C) and CREA (D) on day 10 in each group.


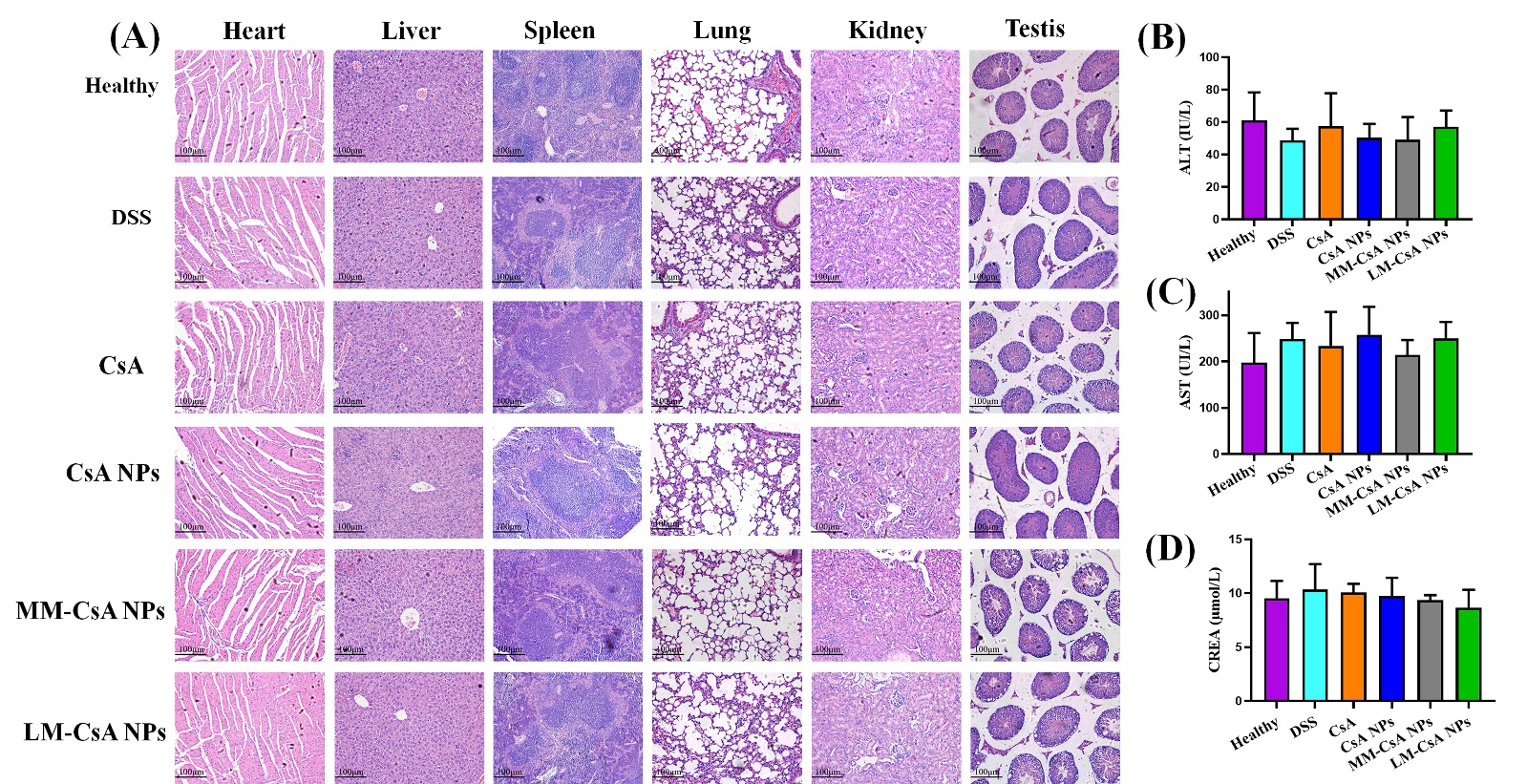


Figure S13, *in vivo* biosafety evaluation of NPs in chronic colitis. H&E-staining of main organs slices from mice in each group (A); and level of ALT (B), AST (C) and CREA (D) on day 10 in each group.
